# Maturation of *Rhodobacter capsulatus* Multicopper Oxidase CutO Depends on the CopA Copper Efflux Pathway and Requires the *cutF* Product

**DOI:** 10.3389/fmicb.2021.720644

**Published:** 2021-09-08

**Authors:** Yavuz Öztürk, Crysten E. Blaby-Haas, Noel Daum, Andreea Andrei, Juna Rauch, Fevzi Daldal, Hans-Georg Koch

**Affiliations:** ^1^Institut für Biochemie und Molekularbiologie, ZBMZ, Faculty of Medicine, Albert-Ludwigs-Universität Freiburg, Freiburg, Germany; ^2^Department of Biology, University of Pennsylvania, Philadelphia, PA, United States; ^3^Biology Department, Brookhaven National Laboratory, Upton, NY, United States; ^4^Department of Biochemistry and Cell Biology, Stony Brook University, Stony Brook, NY, United States; ^5^Fakultät für Biologie, Albert-Ludwigs-Universität Freiburg, Freiburg, Germany

**Keywords:** *Rhodobacter capsulatus*, copper homeostasis, multicopper oxidase, respiratory and photosynthetic growth, copper chaperones, cuproenzyme biogenesis

## Abstract

Copper (Cu) is an essential cofactor required for redox enzymes in all domains of life. Because of its toxicity, tightly controlled mechanisms ensure Cu delivery for cuproenzyme biogenesis and simultaneously protect cells against toxic Cu. Many Gram-negative bacteria contain extracytoplasmic multicopper oxidases (MCOs), which are involved in periplasmic Cu detoxification. MCOs are unique cuproenzymes because their catalytic center contains multiple Cu atoms, which are required for the oxidation of Cu^1+^ to the less toxic Cu^2+^. Hence, Cu is both substrate and essential cofactor of MCOs. Here, we investigated the maturation of *Rhodobacter capsulatus* MCO CutO and its role in periplasmic Cu detoxification. A survey of CutO activity of *R. capsulatus* mutants with known defects in Cu homeostasis and in the maturation of the cuproprotein *cbb*_3_-type cytochrome oxidase (*cbb*_3_-Cox) was performed. This revealed that CutO activity is largely independent of the Cu-delivery pathway for *cbb*_3_-Cox biogenesis, except for the cupric reductase CcoG, which is required for full CutO activity. The most pronounced decrease of CutO activity was observed with strains lacking the cytoplasmic Cu chaperone CopZ, or the Cu-exporting ATPase CopA, indicating that CutO maturation is linked to the CopZ-CopA mediated Cu-detoxification pathway. Our data demonstrate that CutO is important for cellular Cu resistance under both aerobic and anaerobic growth conditions. CutO is encoded in the *cutFOG* operon, but only CutF, and not CutG, is essential for CutO activity. No CutO activity is detectable when *cutF* or its putative Cu-binding motif are mutated, suggesting that the *cutF* product serves as a Cu-binding component required for active CutO production. Bioinformatic analyses of CutF-like proteins support their widespread roles as putative Cu-binding proteins for several Cu-relay pathways. Our overall findings show that the cytoplasmic CopZ-CopA dependent Cu detoxification pathway contributes to providing Cu to CutO maturation, a process that strictly relies on *cutF*.

## Introduction

The redox properties of copper (Cu) make it a suitable cofactor for cuproenzymes that are involved in vital metabolic reactions (Festa and Thiele, 2011; Andrei et al., 2020). However, this inherent property of Cu makes it also very reactive and toxic even at low concentrations by facilitating the production of hydroxyl radicals that attack primarily Fe-S clusters, and by interfering with major biosynthetic pathways, including chlorophyll and *c*-type cytochrome maturation processes (Gaetke and Chow, 2003; Durand et al., 2015; Steunou et al., 2020). Hence, cells have developed sophisticated mechanisms for Cu homeostasis, maintaining the availability of Cu for cuproprotein biosynthesis, while simultaneously preventing Cu toxicity (Osman and Cavet, 2008; Boal and Rosenzweig, 2009; Quintana et al., 2017). During the last decade, the *cbb*_3_-type cytochrome oxidase (*cbb*_3_-Cox) of the facultative phototrophic bacterium *Rhodobacter capsulatus* has been developed into an excellent model for studying cuproprotein biogenesis, and the pathways for Cu detoxification and cuproprotein maturation have been delineated (Ekici et al., 2014; Utz et al., 2019; Andrei et al., 2020). The maturation of the binuclear heme *b*_3_-Cu_B_ catalytic center in subunit CcoN of *cbb*_3_-Cox is a multi-step process that requires the coordinated action of Cu transporters and Cu chaperones to supply Cu^1+^ to *cbb*_3_-Cox (Khalfaoui-Hassani et al., 2016). It begins with the uptake of Cu^2+^ by the major facilitator superfamily (MFS) protein CcoA (Ekici et al., 2012; Zhang et al., 2019). Next, intracellular Cu^2+^ is reduced to Cu^1+^ by the Cu reductase CcoG (Marckmann et al., 2019) and then conveyed via the cytoplasmic chaperone CopZ to the P_1__B_-type ATPase CcoI for Cu^1+^ translocation into the periplasm (Utz et al., 2019). Especially under low Cu availability, Cu is inserted into *cbb*_3_-Cox by sequential interactions of the periplasmic Cu chaperones SenC and PccA (Lohmeyer et al., 2012; Trasnea et al., 2018).

In many bacteria, excess Cu is sensed by CueR-like transcription factors, which regulate the expression of Cu tolerance genes including *copA*, *copZ*, and *cueO* (Outten et al., 2000; Stoyanov et al., 2001; Changela et al., 2003; Peuser et al., 2011; Philips et al., 2015). Cu-exporting P_1__B_-type ATPases such as CopA represent the central component of the Cu-efflux pathway in most Gram-negative bacteria (Petersen and Moller, 2000; Rensing et al., 2000; Argüello et al., 2013). Like CcoI, CopA receives Cu from the cytoplasmic chaperone CopZ and exports it in an ATP-dependent step to the periplasm (Gonzalez-Guerrero and Arguello, 2008; Utz et al., 2019). In the periplasm, laccase-like multicopper oxidases (MCOs), such as CueO of *Escherichia coli* and CutO of *R. capsulatus*, play a crucial role in Cu detoxification by oxidizing Cu^1+^ to the less toxic Cu^2+^ (Cha and Cooksey, 1991; Grass and Rensing, 2001; Outten et al., 2001; Wiethaus et al., 2006; Achard et al., 2010; Rowland and Niederweis, 2013; Granja-Travez and Bugg, 2018).

**FIGURE 1:**
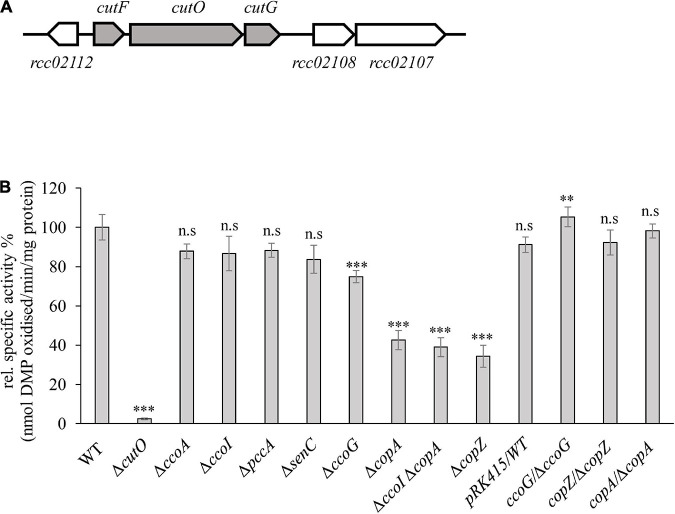
Genetic organization and activity of the multi-copper oxidase (MCOs) CutO in *Rhodobacter capsulatus*. **(A)** Genetic organization of the *cutFOG* operon in *R. capsulatus*. *CutO* codes for the multicopper oxidase (*rcc02110*), and *cutF* (*rcc02111*) and *cutG* (*rcc02109*) for largely uncharacterized proteins. The open reading frame *rcc02112* encodes a putative glyoxalase/bleomycin resistance protein/dioxygenase. The open reading frames *rcc02108* and *rcc02107* encode hypothetical proteins with unknown function. **(B)** CutO activity of different *R. capsulatus* mutants deficient in *cbb*_3_-Cox assembly and Cu homeostasis. Periplasmic fractions were isolated from the indicated strains, grown in magnesium–calcium, peptone, yeast extract (MPYE) medium supplemented with 10 μM CuSO_4_. 50 μg periplasmic soluble protein was used in the 2,6-DMP assay and absorbance was measured at 468 nm. The activity of wild-type (WT) was set to 100% and the relative activities of the indicated strains were calculated. The activities of mutant strains that showed significantly reduced CutO activity were also tested after complementation with pRK415-borne copies of the respective genes. WT containing the pRK415 vector served as a control. Three independent experiments were performed with three technical replicates and the error bars reflect the standard deviation (*n* = 9). Statistical analyses were performed with the Satterthwaite corrected two-sided Student *t*-test, using the activity of the WT as reference. (^∗^) refers to *p*-values ≤ 0.05; (^∗∗^) to *p*-values ≤ 0.01, and (^∗∗∗^) to *p*-values ≤ 0.001.

Laccase-like MCOs are unique enzymes because they require Cu ions in their catalytic centers for Cu^1+^ oxidation, hence Cu serves as a cofactor and a substrate for these enzymes. These Cu oxidases are typically monomeric proteins that consist of three cupredoxin domains and contain four Cu atoms in the catalytic site. These Cu ions are discriminated by distinct spectroscopic properties and referred to as T1 Cu, T2 Cu and the binuclear T3 Cu (Bello et al., 2012). Substrates bind to the T1 Cu, also referred to as blue Cu, which shuttles electrons to a trinuclear Cu site (TNC), composed of the T2 Cu and the two T3 Cu ions (Bello et al., 2012). In addition to the four Cu ions in the catalytic site, MCOs can also bind additional Cu via their methionine-rich segments (MRS), and at least three additional Cu are bound to the MRS in *E. coli* CueO (Singh et al., 2011). However, the length and sequence of MRS are variable between MCOs from different species (Fernandes et al., 2007; Djoko et al., 2008; Sakuraba et al., 2011). *R. capsulatus* CutO exhibits similarity (25% identity) to CueO of *E. coli*, and in particular, all Cu binding motifs are completely conserved (Wiethaus et al., 2006). CutO is encoded in the tricistronic *cutFOG* operon (*rcc02111*, *rcc02110*, and *rcc02109*) (Figure 1A), and all three genes (initially called *orf635-cutO-cutR*) are required for Cu tolerance (Wiethaus et al., 2006). The transcription of *cutO* strictly depends on the promoter upstream of *cutF* (Wiethaus et al., 2006), and the expression of *cutO* and *cutG* is affected by Cu availability, unlike *cutF* expression (Rademacher et al., 2012; Selamoglu et al., 2020). However, the mechanisms of this Cu-dependent transcriptional regulation are largely unknown. Initially, it has been suggested that CutG acts as a transcriptional repressor in the absence of Cu (Wiethaus et al., 2006). The product of *cutG* is homologous to the Cu-thiol oxidoreductase CopG of *Pseudomonas aeruginosa* (42% identity and 68% similarity) and is widely distributed among Gram-negative bacteria. It is frequently present in Cu resistance-conferring gene clusters encoding CueO, CopA, and CusCBA (Monchy et al., 2006; Wiethaus et al., 2006; Behlau et al., 2011; Marrero et al., 2012). *P. aeruginosa* CopG contains a cysteine-bridged tetranuclear Cu cluster and contributes to Cu resistance under anaerobic conditions via its Cu oxidoreductase activity (Hausrath et al., 2020). In addition to its enzymatic activity, CopG is proposed to be involved in Cu transfer reactions (Hausrath et al., 2020).

Unlike CutG, CutF-like proteins are detected by BLASTp in only a small subset of bacterial genomes. Searching with CutF against the non-redundant protein sequence database with default parameters identifies similar proteins in only 10 genomes, all from *Rhodobacteraceae* (see section “Results”). CutF from *R. capsulatus* encodes a predicted small protein of 118 amino acids, and has a putative Sec signal sequence, suggesting that CutF is translocated to the periplasm. Sequence alignment of CutF homologs shows a conserved CXXXC motif in its central part that could ligate Cu. *cutF* expression does not seem to be regulated by Cu (Rademacher et al., 2012), and a *cutF* translation product was not detected in comparative cuproproteome analyses of *R. capsulatus*, despite large differential variations of CutO- and CutG-levels under Cu-supplemented or Cu-depleted growth conditions (Selamoglu et al., 2020). These observations suggest that CutF might be a low abundance protein of unknown function, potentially required for the production of active CutO. Furthermore, in the light of the dual role of Cu as an essential cofactor and also as a substrate of MCOs, it is unclear whether the Cu cofactor required for forming the catalytic center and the substrate Cu^1+^ that is converted to Cu^2+^ by the catalytic center, are provided by the same Cu-delivery pathway. In the current study, we performed a rule-based bioinformatic search combined with profile hidden Markov Models (HMMs) to identify CutF-like proteins encoded by available proteobacterial genomes and tested the role of CutF for CutO activity, and the source of catalytic Cu and substrate Cu for CutO assembly and activity were investigated.

## Materials and Methods

### Bacterial Strains and Growth Conditions

The bacterial strains and plasmids used in this study are described in Supplementary Table 1. *R. capsulatus* strains were grown under respiratory (Res) or photosynthetic (PS) conditions on magnesium–calcium, peptone, yeast extract (MPYE) enriched medium (Daldal et al., 1986), or Sistrom’s minimal medium A (Med A; Sistrom, 1960), supplemented with kanamycin, spectinomycin, gentamycin or tetracycline as appropriate (10, 10, 1 or 2.5 μg per mL, respectively) at 35°C. For PS growth, cultures were incubated under saturating light intensity in anaerobic jars containing H_2_ and CO_2_ generating gas packs (Becton Dickinson and Co.) (Jenney and Daldal, 1993). For protein production of arabinose-inducible genes in *R. capsulatus*, liquid media were supplemented with 0.5% L-arabinose (L-ara) at OD_685_ of 0.5–0.6 and further growth for 6 h. *E. coli* strains were grown on lysogeny broth medium (LB; Bertani, 1951), containing ampicillin, kanamycin, or tetracycline (at 100, 50, or 12.5 μg per mL, respectively) as appropriate.

### Copper-Sensitivity Assays and NADI Staining on Plates

The growth of *R. capsulatus* strains in the presence of different CuSO_4_ concentrations was monitored using spot assays (Utz et al., 2019). Strains were grown semi aerobically overnight to an OD_685_ of ∼ 0.9 and cell counts were determined based on OD_685_ of 1.0 = 7.5 × 10^8^ cells/mL. For each strain, 1 × 10^8^ cells were resuspended in 400 μL medium and subsequently serially diluted in a 96-well plate. Dilutions ranging from 10^0^ to 10^–7^ were spotted on MPYE plates containing different concentrations of CuSO_4_ by a 48 pin replica plater. The plates were incubated under the Res and Ps conditions for approx. 2 and 3 days, respectively, before scoring the data.

The *in vivo cbb*_3_-Cox activity of *R. capsulatus* colonies was visualized qualitatively using the “NADI” staining solution made by mixing 1:1 (v/v) ratio of 35 mM α-naphtol and 30 mM *N*, *N*, *N*′, *N*′ –dimethyl-*p*-phenylene diamine (DMPD) dissolved in ethanol and water, respectively (Koch et al., 1998b).

### Molecular Genetic Techniques

#### Chromosomal Inactivation of *cutFOG* and *copA* in a Δ*ccoI* (CW2) Strain

Chromosomal knock-out alleles of *cutG* and *cutF* genes were obtained by interposon mutagenesis using the Gene Transfer Agent (GTA; Yen et al., 1979; Daldal et al., 1986) and in-frame markerless chromosomal deletion method, respectively (Brimacombe et al., 2013). A Δ(*cutG*:*Gm*) deletion-insertion allele, carried by the conjugative plasmid pRK415 (Ditta et al., 1985) in the *R. capsulatus* GTA overproducer strain Y262 was used to inactivate the chromosomal copy of *cutG* in Δ*cutO* and Δ*cutFO* (YO-ΔcutFO) strains (Selamoglu et al., 2020) to obtain the Δ*cutOG* double (Δ*cutO*Δ*cutG*) and the Δ*cutFOG* (Δ*cutF*Δ*cutO*Δ*cutG*) triple mutants (Supplementary Table 1). The in-frame, markerless chromosomal deletion method was used for creating a Δ*cutF* in-frame deletion allele, containing only its first four and last four codons, carried by the suicide plasmid pZDJ (pYO-Δ02111Su) in *E. coli* strain S17-1 (Selamoglu et al., 2020). This plasmid was conjugated into the Δ*cutO* strain to obtain the Δ*cutFO* double mutant. The transconjugants were selected for Gm^r^ and counter-selected using sucrose, which is toxic in the presence of *sacB* carried by the pYO-Δ02111Su plasmid. After two passages in non-selective liquid MedA, colonies carrying a chromosomal deletion of *cutF* were selected for their ability to grow under Res conditions in the presence of 10% sucrose due to second homologous recombination eliminating the chromosome-integrated copy of pYO-Δ02111Su (Brimacombe et al., 2013; Kuchinski et al., 2016). A few of these sucrose-resistant and Gm^s^ colonies were screened by colony PCR to detect the in-frame deletions, and amplified fragments were confirmed by DNA sequencing. The Δ*ccoI*Δ*copA* double mutant was obtained by using the pRK-CopA2:Kan plasmid (Ekici et al., 2014) carrying the Δ(*copA:kan*) allele in the GTA overproducer Y262 strain. GTA particles isolated from this strain were used with the Δ*ccoI* strain for GTA-mediated interposon mutagenesis as above (Yen et al., 1979; Daldal et al., 1986).

#### Cloning of *cutFOG* Operon With Its Native Promoter and Construction of Epitope-Tagged Protein Variants

Standard molecular genetic techniques were performed as described previously (Koch et al., 1998a; Sambrook and Russel, 2001). The tagged version of *cutFOG* genes (*cutF*_Strep_*O*_Flag_*G*_MycHis_ or *cutF*_Flag_*O*_Flag_*G*_MycHis_) were cloned while maintaining the integrity of the operon, including its 526 bp upstream (promoter) and 466 bp downstream (transcriptional terminator) DNA regions using appropriate primers (Supplementary Table 2). The DNA fragments encoding the N-terminally or C-terminally tagged version of the corresponding proteins were amplified by PCR using the wild-type (WT) MT1131 genomic DNA as a template. The PCR primers used for the NEBuilder^R^ HiFi assembly cloning method (NEB Lab, United States) contained 20 bp long 5′ overlapping regions between the vector arms and the gene fragments, and after amplifications, the PCR products were purified by Qiagen PCR purification kit (Qiagen, Hilden, Germany). The quantity and quality of the amplified DNA fragments were checked via a nanodrop spectrophotometer and agarose gel electrophoresis, respectively, and used in the assembly reactions. HiFi assembly master mix (NEBuilder^R^) was used in a single step reaction to assemble the 526 bp upstream fragment, *cutF*_Strep_ (or *cutF*_Flag_), *cutO*_Flag_, *cutG*_MycHis_, 466 bp downstream fragment, and the conjugative plasmid pRK415 digested with *Kpn*I and *Xba*I restriction enzymes, according to the NEB protocol. In each case, the total amount of DNA fragments used was ∼ 0.4 – 0.5 pmoles, and the vector to insert ratio was ∼ 1:2 to 1:3. The samples were incubated in a thermocycler at 50°C for 60 min, and 4 μL of the assembly reaction was transformed to a chemically competent *E. coli* HB101 strain. Two versions of *cutF*_Strep_ were constructed, one containing the tag at its C-terminus with a Gly-Gly-Ser-Ala (GGSA) linker, and the other containing the tag at its N-terminus after its signal peptide cleavage site with a GGSA linker. A similar N-terminally tagged version of *cutF*, replacing its Strep tag with a Flag tag was also constructed to yield *cutF*_*N*__–Flag_. The *cutO*_Flag_ and *cutG*_MycHis_ were cloned by adding their tags at the C-termini of all constructs. The constructed conjugative plasmids pRK-cutFOG1 (*cutF*_C–__Strep_*O*_Flag_*G*_MycHis_) pRK-cutFOG2 (*cutF*_*N–*__Strep_*O*_Flag_*G*_MycHis_) and pRK-cutFOG3 (*cutF*_*N–*__Flag_*O*_Flag_*G*_MycHis_) were confirmed by DNA sequencing. The correct clones were conjugated into the *R. capsulatus ΔcutFOG* strain via triparental crosses (Supplementary Table 1).

#### Construction of CutO, CutF, and CutG Mutant Derivatives

Two mutant versions of CutO were constructed by substituting Cys473 to Ala (CutO_C__473__A_, leading to the loss of the T1 Cu site), and by deleting the 41 amino acid long methionine rich MDHGAMDHSATPMQGMDGMADMMSLPGMAEMHAAME GGLSM sequence between the domains 1 and 2 of CutO (CutO_Δ__MRS_) using the mutagenic primers cutF(EP)-F, cutO(C473A)-R, cutGopr-F, and cutTer-R for CutO_C__473__A_, cutF(EP)-F, cutOdelMRS-R, cutOdelMRS-F, cutOopr-R, cutGopr-F and cutTer-R for CutO_Δ__MRS_ (Supplemetary Table 2), using the *cutFOG* operon with its native promoter and terminator regions. The amplified fragments were assembled into the *Kpn*I-*Xba*I digested pRK415 vector as above. For these mutations, pRK-cutFO_Flag_G plasmid carrying the CutFO_Flag_G variant was used as a template. This plasmid was constructed by using the primers cutF(EP)-F, cutOopr-R, cutOopr-F, and cutTer-R (Supplementary Tables 1, 2) as described above.

Substitution of two conserved Cys residues in CutF by Ala (C_69_XXXC_73_ to A_69_XXXA_73_) and truncation of C-terminal Proline-rich region (ΔP_109_EPEGPPPRL_118_) were constructed on pRK-cutFOG3 (*cutF*_*N–*__Flag_*O*_Flag_*G*_MycHis_) by using the mutagenic primers cutF(EP)-F, cutF(C-A)-R, cutF(C-A)-F and cutTer-R for CutF_C–A_, cutF(EP)-F, cutF(del-Cter)-R, cutF(del-Cter)-F and cutTer-R for CutF_Δ__C–ter_ (Supplementary Table 2). The amplified fragments were assembled into the *Kpn*I-*Xba*I digested pRK415 vector as above.

Substitution of three conserved Cys residues of CutG to Ala (C_40_XC_42_C_43_ to A_40_XA_42_A_43_) and three Met residues to Ala (M_120_X_(__6__)_M_127_ to A_120_X_(__6__)_A_127_ of CutG) were constructed on pRK-cutFOG3 (*cutF*_*N–*__Flag_*O*_Flag_*G*_MycHis_) by using the mutagenic primers cutF(EP)-F, cutG(C-A)-R, cutG(C-A)-F and cutTer-R for CutG_C–A_, cutF(EP)-F, cutG(M-A)-R, cutG(M-A)-F and cutTer-R for CutG_M__–A_ (Supplementary Table 2). Assembly of the fragments was performed as described above.

#### Cloning of pRS1-*cutF* for *in vivo* and *in vitro* Expression

The ORF of *cutF* was cloned into the plasmid pRS1 (Jauss et al., 2019) for *in vivo* and *in vitro* expression. The vector pRS1 was linearized by using the pRS1-F and pRS1-R primers at the start codon ATG. The amplification mixture containing the linear form of pRS1 was digested with *Dpn*I to remove its circular form used as a PCR template. The *cutF*_N–Flag_ including a 20 bp overlapping sequence of pRS1 vector was amplified from pRK-cutFOG3 by using the primers pRS1cutF-F and pRS1cutF-R (Supplementary Table 2) and Q5^®^ High-Fidelity DNA Polymerase (NEB Lab, MA, United States). The HiFi assembly (NEBuilderR) and transformation procedures similar to those described above were used for this construction, except that the 4 μL of the assembly reaction mixture was transformed to chemically competent *E. coli* NEB^®^ 5-alpha and C43(DE3) strain, selecting for Amp^r^ colonies. The resulting plasmid pRS1-cutF was confirmed by DNA sequencing.

#### Total RNA Isolation and Transcription Analysis by RT-PCR

Semi-aerobic (Res grown) cultures of *R. capsulatus* in MPYE medium with (10 μM CuSO_4_) and without Cu addition were grown to mid-log phase (OD_685_ 0.5–0.8). Total RNA was isolated from ∼1 × 10^9^ cells thus obtained using the Qiagen RNeasy mini kit (Qiagen, Hilden, Germany), and digested with DNase I for 30 min at 25°C in the presence of RNase inhibitor RNasin. ∼120–180 ng of total RNA was used in RT-PCR reactions with the One-Step RT-PCR kit (Qiagen, Hilden, Germany). Transcriptional features of the *cutFOG* operon were analyzed using the cutFO(rtPCR)-F/cutFO(rtPCR)-R primer pairs covering 400 bp of *cutFO*, and the cutOG(rtPCR)-F/cutOG(rtPCR)-R primer pairs covering 500 bp of *cutOG*, and the 16SrRNA-F and 16SrRNA-R primers provided an internal control for amplification of a 450-bp region of *R. capsulatus* 16S rRNA gene (Supplementary Table 2; Ekici et al., 2014). Control reactions omitted the RNA template from the reaction mixture and the amplified RT-PCR products were separated using 2% agarose gels.

### Biochemical and Biophysical Techniques

#### Membrane Preparation for SDS-PAGE and TMBZ Staining

Cytoplasmic membrane fractions and supernatant fractions (soluble cytoplasmic and periplasmic proteins) were prepared by using 50 or 250 mL of *R. capsulatus* cultures grown overnight under Res conditions in MPYE. Cells were broken by using either a French pressure cell at 18,000 lb/inch^2^ or an Emulsiflex C3 homogenizer (Avestin, Ottawa, ON, Canada) at 10,000 lb/inch^2^ as described earlier (Gray et al., 1994; Knupffer et al., 2019). Membranes were resuspended in 400 μL of 50 mM triethanolamine acetate (pH 7.5), 0.5 mM phenylmethylsulfonyl fluoride (PMSF), 1 mM EDTA, and 1 mM dithiothreitol, and the protein concentrations were determined by using a modified Lowry assay (Noble and Bailey, 2009).

#### Multicopper Oxidase Activity

The oxidation of 2,6-dimethoxyphenol (2,6-DMP) by the periplasmic fraction (20 or 50 μg total protein) of *R. capsulatus* cells was monitored either continuously with a TIDAS 100 spectrophotometer for 15 min or with Ultrospec 3,100 pro reader for endpoint measurement at 468 nm. The molar concentration of oxidized 2,6-DMP was calculated using ε = 14,800 M^–1^ cm^–1^ of oxidized 2,6-DMP (Solano et al., 2001).

The periplasmic fraction was isolated following published protocols (McEwan et al., 1984; Trasnea et al., 2016). In brief, bacterial cultures (50 mL) were grown overnight on MPYE under Res or PS conditions in the presence or absence of supplementary CuSO_4_, as specified in the legends to the figures. The entire culture was harvested and washed at 4°C with 12 mL of 50 mM Tris–HC1 pH 8.0, the final pellet resuspended to a concentration of 10 mL/g of wet weight in the SET buffer (0.5 M sucrose, 1.3 mM EDTA, 50 mM Tris–HC1 pH 8.0), incubated with 600 μg lysozyme/mL at 30°C for 60 min, and spheroplasts formation was monitored by microscopy (Supplementary Figure 1). The suspension was sedimented at 4°C by centrifugation at 13.000 rpm for 30 min. The supernatant (periplasmic fraction) was stored on ice while the pellet was resuspended in a total volume of 1.5 mL of SET buffer in 2 mL eppendorf tubes. This suspension was sonicated on ice with a Dawe Soniprobe (United Kingdom) four times with 15 s sonication and 30 s break at 150 W and then centrifuged at 5.000 rpm for 10 min. The pellet was discarded and the supernatant was further centrifuged at 45.000 rpm for 90 min. The supernatant (cytoplasmic fraction) together with the last pellet resuspended in 100 μL of ICM buffer (membrane fraction) were stored at −80°C.

#### Immune Detection and Heme Staining

For immune-detection following SDS- or BN-PAGE, proteins were electro-blotted onto nitrocellulose (GE Healthcare, Germany) or PVDF Immobilon-P (GE Healthcare, Germany) membranes, respectively, and antibodies against the Flag-, His- or Myc-tags (clone 9E10) were purchased from either Sigma or Millipore (Temecula, CA, United States). Heme staining was performed as described earlier (Koch et al., 1998a). For this purpose, 16.5% Tris–Tricine SDS-PAGE were used (Schagger and Von Jagow, 1987). Samples were denatured in SDS-loading buffer for 10 min at 95°C for soluble proteins, 20 min at 37°C for membrane-bound proteins, and following electrophoresis, the *c*-type cyts revealed by their peroxidase activities using 3,3′, 5, 5′ tetramethylbenzidine (TMBZ) and H_2_O_2_ (Thomas et al., 1976).

#### *In vitro* Synthesis of CutF

An *in vitro* coupled transcription-translation system was used to synthesize CutF. Cytosolic translation factors (CTF) and high-salt washed ribosomes were used in 25 μL aliquots, as described before (Hoffschulte et al., 1994; Koch et al., 1999). A final concentration of 11 mM MgAc was used in all samples, a control sample lacking ribosomes was included, and the radioactive labeling mix containing ^35^S-Methionine/^35^S-Cysteine was purchased from Hartmann Analytic (Braunschweig, Germany). The reaction mixture was prepared on ice and then incubated for 30 min at 35°C with gentle shaking. At the end of the synthesis, the *in vitro* reaction was mixed with 1:1 10% TCA, precipitated for 40 min on ice, the samples were pelleted in a precooled table-top centrifuge at 13,700 × *g* for 18 min. The pellet was further resuspended in 30 μL TCA, mixed with a loading dye prepared as described earlier (Steinberg et al., 2020), and subsequently denatured at 37°C for 20 min. The samples were subjected to a 5–15% SDS-PAGE, and the separated proteins were visualized by phosphorimaging.

### Phylogenomic Analyses

#### Identification of CutF-Like Proteins

Because of the relatively small size (118 amino acid residues) and poor sequence conservation, a rule-based approach was used to identify CutF-like protein sequences. Genes within a 10-gene window surrounding CutO-encoding homologs (defined as containing either PF00394 or PF07731 or both) from publicly available proteobacterial genomes were collected using the genomic context tool EFI-GNT (Gerlt, 2017; Zallot et al., 2019). Gene neighbors were then filtered based on the following criteria: the encoded protein is smaller than 170 amino acids, does not match to a known Pfam domain, contains a predicted signal peptide, a CXXXC sequence motif (where X is any amino acid), and the PP sequence motif. The resulting protein sequences were aligned with MUSCLE (Madeira et al., 2019), viewed with Jalview (Waterhouse et al., 2009), and sequences were removed based on manual inspection of the CXXXC motif alignment. The multiple sequence alignment thus obtained was used to search for similar proteins via jackhammer (Potter et al., 2018) against the Proteobacterial Reference Proteome set in the UniProt database (The UniProt Consortium, 2019) with 11 iterations at which point no additional sequences were identified. Any sequence matching to a Pfam domain was removed, resulting in a total of 2,938 sequences that we refer to as CutF-like proteins.

#### Copper-Oxidase Sequence Similarity Network

Bacterial reference proteomes from the UniProt database were searched for proteins that contained the three Cu-oxidase domains PF00394, PF07731, and PF07732, resulting in 4,040 sequences. The EFI-Enzyme Similarity Tool (EFI-EST^1^; Gerlt et al., 2015) was used to build the similarity network with an alignment score of 100, and nodes were collapsed at a similarity of 90%. The network thus built was visualized with Cytoscape (Shannon et al., 2003) and nodes were clustered using the yFiles organic layout.

#### Copper Oxidase Phylogenetic Reconstruction

Proteobacterial Cu oxidase-like proteins from the sequence similarity network were extracted and mapped to UniRef90 in the UniProt database (Suzek et al., 2015). Phylogenetic analysis was performed using the CIPRES web portal (Miller et al., 2010) with MAFFT on XSEDE (v. 7.305) for the sequence alignment (Katoh and Standley, 2013) and FastTree with 1,000 bootstrap replicates (Price et al., 2010). Branches with less than 50% bootstrap support were deleted.

## Results

### CutO Activity Is Largely Independent of the Cu Supply System for *cbb*_3_-Cox Assembly, but Partly Relies on the CopZ-CopA Cu Export Pathway

Maturation of the cuproprotein *cbb*_3_-Cox depends on a complex Cu delivery chain that involves at least six proteins, which shuttle Cu from the extracellular space into the cytosol and back to the periplasm prior to Cu insertion into the catalytic subunit CcoN (Andrei et al., 2020). The complexity of this Cu supply chain raises the question of whether it is dedicated solely to *cbb*_3_-Cox assembly or also used at least partly for the maturation of other extracytoplasmic cuproproteins, such as the periplasmic CutO protein. CutO activities of mutant strains (Δ*ccoA*, Δ*ccoG*, Δ*copZ*, Δ*ccoI*, Δ*senC*, and Δ*pccA*) that are deficient in known proteins required for Cu delivery to *cbb*_3_-Cox were determined using the 2,6-DMP assay with periplasmic extracts of the mutant strains (Solano et al., 2001). The specific activity of the wild-type (15 nanomoles of 2,6-DMP oxidized/min/mg of protein) was set to 100% and the specific activity in the mutants was determined (Figure 1B). The single Δ*cutO* mutant served as a control and showed no 2,6-DMP oxidation, indicating that this assay monitors exclusively CutO mediated multi copper oxidase activity in *R. capsulatus* periplasmic extracts. In comparison to the wild-type activity, no significant difference in the Δ*ccoA* strain that lacks the MFS-type Cu importer was seen. Mutant strains lacking the two periplasmic chaperones SenC and PccA, which are required for *cbb*_3_-Cox assembly at low Cu concentrations, or CcoI, the Cu-exporting P_1__B_-type ATPase that is essential for *cbb*_3_-Cox biogenesis (Kulajta et al., 2006; Lohmeyer et al., 2012; Trasnea et al., 2018), also showed no detectable reduction of CutO activity. A small but reproducible reduction of CutO activity (approx. 25%) was observed in the absence of the cupric reductase CcoG (Marckmann et al., 2019), which was fully restored to wild type activity by a plasmid-borne copy of *ccoG* (Figure 1B). CcoG reduces Cu^2+^ to Cu^1+^, which is then bound by the chaperone CopZ or the P_1__B_-type ATPases CcoI and CopA (Gonzalez-Guerrero and Arguello, 2008; Utz et al., 2019). In contrast to CcoI, CopA is dispensible for *cbb*_3_-Cox biogenesis, but is required for Cu detoxification (Ekici et al., 2014), which in *R. capsulatus* also involves CcoG and CopZ (Marckmann et al., 2019; Utz et al., 2019). Therefore, the CutO activities of Δ*copA* and Δ*copZ* single, as well as a Δ*copA*Δ*ccoI* double mutants were tested. These assays showed similar residual activities (about 60% reduction compared to wild-type) for both the single and double Δ*copA* mutants, and the single *copZ* mutant (Figure 1B). The reduced MCO activities in the Δ*copA* and Δ*copZ* single mutants were fully restored by providing the respective genes in *trans* (Figure 1B). Considering that all strains grew well in the presence of 10 μM Cu, overall data showed that CutO production is independent of the *cbb*_3_-Cox maturation machinery, except for CcoG and CopZ, and relies partly on the CopZ-CopA dependent Cu detoxification pathway. As none of the deletion mutants examined resulted in a complete loss of CutO activity, part of the Cu supply to CutO may be provided by other proteins, or may come directly from the periplasm.

### Genes of the Multicopper Oxidase Operon *cutFOG* Are Important Determinants of Cu Resistance Under Aerobic and Anaerobic Growth Conditions

**FIGURE 2:**
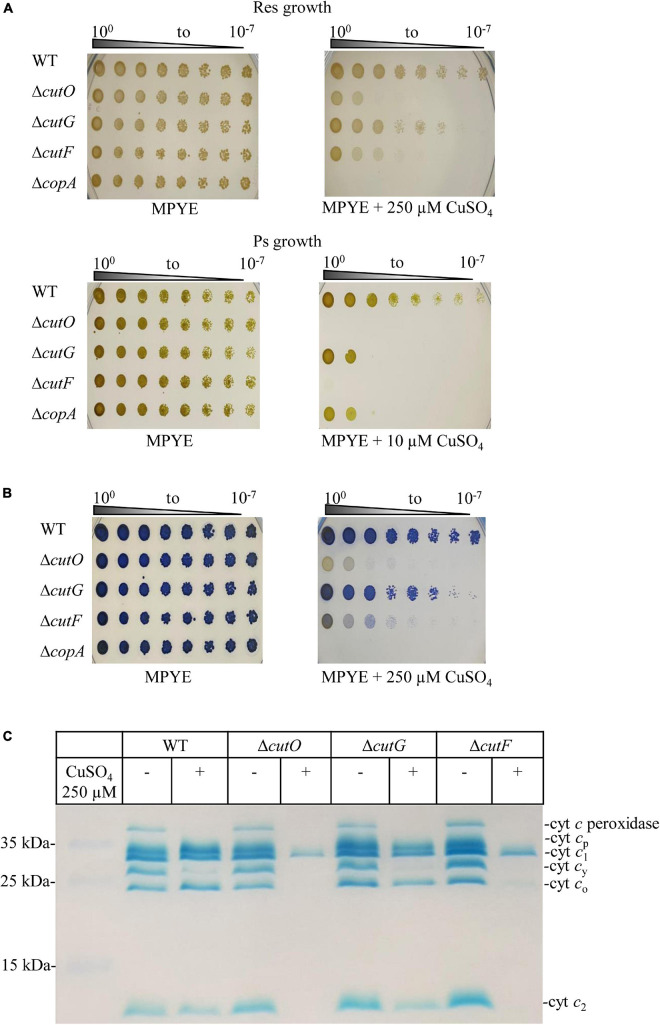
Copper (Cu) sensitivity assay and cytochrome *c* profiles of the *cutF*, *cutO*, and *cutG* knockout strains of *R. capsulatus*. **(A)** WT and the indicated knockout strains were serially diluted and growth was tested on MPYE agar plates supplemented with 10 or 250 μM CuSO_4_ for photosynthetic and respiratory growth, respectively, and incubated under appropriate growth conditions for 2–3 days. As a control, the Δ*cop*A strain lacking the Cu-exporting P_1__B_-type ATPase CopA was included. **(B)** The *cbb*_3_-Cox activities of the indicated strains grown under respiratory conditions on MPYE + 250 μM CuSO_4_ were determined by the NADI reaction. The blue dye indophenol blue is formed within 45 s in the presence of active *cbb*_3_-Cox. No or very low cytochrome *c* oxidase activities were observed for the Δ*cutO* and Δ*cutF* strains in the presence of 250 μM Cu. **(C)** The *c*-type cytochrome profiles of mutant strains grown in the presence and absence of supplemented CuSO_4_ were determined by heme-staining of isolated membranes and after separation on 16.5% SDS-PAGE. Cyt *c*_p_ and cyt *c*_o_ correspond to the subunits of *cbb*_3_-Cox, cyt *c*_1_ is the subunit of cyt *bc*_1_ complex, and cyt *c*_y_ and cyt *c*_2_, correspond to membrane-bound and soluble electron carriers, respectively.

The physiological roles of the *cutFOG* gene products were analyzed by creating individual Δ*cutF*, Δ*cutO*, and Δ*cutG* knock-out mutants and by measuring their growth at different Cu concentrations under both respiratory (Res) and anaerobic-photosynthetic (Ps) conditions. Supplemental Cu concentrations ranging from 5 to 600 μM and 5 to 75 μM CuSO_4_ were used for Res- and Ps-growth, respectively (Supplementary Figures 2, 3). As controls, the Δ*copA* and Δ*copZ* strains were included, and their Cu sensitivity profiles matched previously reported observations (Utz et al., 2019; Supplementary Figure 2). Under Res-condition the wild type grew up to 500 μM Cu, but growth impairment of the Δ*cutO* mutant started already at 250 μM CuSO_4_ (Figure 2A) and complete inhibition was observed at 400 μM CuSO_4_ (Supplementary Figure 2). The Δ*cutF* strain showed a similar Cu sensitivity, although slightly less pronounced than in the Δ*cutO* mutant (Figure 2A). In contrast, the Δ*cutG* strain was less Cu sensitive than the Δ*cutO* and Δ*cutF* strains (Figure 2A), and complete growth inhibition was only observed at 500 μM CuSO_4_ (Supplementary Figure 2). Despite the widespread distribution of CutG-like Cu resistance proteins and the more limited occurrence of CutF-like proteins (see below), *R. capsulatus* CutF appears to be as crucial for Cu detoxification as the multicopper oxidase CutO. However, the Δ*cutO* and Δ*cutF* mutants tolerated higher Cu concentrations than the Δ*copA* strain under Res growth condition (Figure 2A). This observation is likely due to the accumulation of cytosolic Cu^1+^ in the *R. capsulatus* Δ*copA* strain (Ekici et al., 2014; Utz et al., 2019), which might be more toxic than the accumulation of Cu^1+^ in the periplasm when CutO or CutF are absent.

One of the major targets of excess Cu in the periplasm is the cytochrome c maturation (Ccm) pathway (Sanders et al., 2005; Durand et al., 2015; Verissimo et al., 2017). Due to the presence of a quinol oxidase, the Ccm pathway is not essential for growth of *R. capsulatus* under Res conditions (Koch et al., 1998a; Swem and Bauer, 2002). However, the Ccm pathway is required for Ps-growth, and the Δ*cutF*, Δ*cutO*, and Δ*cutG* knock-out strains were therefore also tested under PS conditions. In agreement with the inhibitory effect on *c*-type cytochrome biogenesis (Durand et al., 2015), the *cutO* and *cutF* deletions strains were highly Cu sensitive under PS conditions (Figure 2A and Supplementary Figure 3). In comparision to the wild type, which grew up to 75 μM Cu, the Δ*cutG* and Δ*copA* strains were also more Cu sensitive, but still showed partial growth at 10 μM Cu (Figure 2A and Supplementary Figure 3). Although the Ccm pathway is not essential under Res condition, it is required for the activity of *cbb*_3_-Cox, as this enzyme contains two membrane-bound *c*-type cytochrome subunits (Gray et al., 1994; Koch et al., 1998a). Hence, the inhibition of the Ccm pathway by Cu accumulation in the periplasm was further probed by determining the *cbb*_3_-Cox activity using the NADI staining under Res conditions. All three mutant strains showed a NADI-positive response on plates without Cu supplementation, indicating that the absence of the *cutFOG* genes *per se* did not influence *cbb*_3_-Cox activity. However, in the presence of 250 μM Cu, the Δ*cutO* strain did not turn blue when the NADI-reagent was applied, indicating the lack of *cbb*_3_-Cox activity (Figure 2B). A reduced blue coloring was also observed in the Δ*cutF* strain, unlike the Δ*cutG* strain that was comparable to the wild-type in response to NADI staining (Figure 2B). This was further validated by performing tetramethylbenzidine (TMBZ)-mediated heme staining on membranes isolated from the deletion strains grown by respiration in the presence or absence of 250 μM Cu (Figure 2C and Supplementary Figure 4). Membranes of the Cu-supplemented wild-type strain showed reduced amounts of the membrane-bound electron donor cytochrome *c*_y_ and cytochrome *c* peroxidase (29 and 37 kDa, respectively), but no major difference was observed in the amounts of CcoO and CcoP, the two *c*-type cytochrome subunits of *cbb*_3_-Cox. There was also no difference in the amounts of the cytochrome *c*_1_ subunit of the *bc*_1_ complex and the soluble electron donor cytochrome *c*_2_. In contrast, membranes of the Cu supplemented Δ*cutO* and Δ*cutF* mutants exhibited a different cytochrome profile than the wild type and the Δ*cutG* mutant. In the absence of CutO or CutF, only cytochrome *c*_1_ was detectable and the cytochrome subunits CcoO and CcoP of *cbb*_3_-Cox were absent, while in the absence of CutG a cytochrome *c* profile similar to that of wild-type cells was observed (Figure 2C).

In summary, these data demonstrated that the multicopper oxidase CutO and the functionally uncharacterized putative protein CutF are important determinants for preventing Cu toxicity and inhibition of cytochrome biogenesis in the bacterial periplasm. In contrast, although widely conserved among bacteria, CutG does not seem to play a major role in Cu detoxification in *R. capsulatus*, suggesting that its function is dispensable or redundant with other *R. capsulatus* proteins.

### CutO Activity Is Strictly Dependent on CutF, While CutG Is Partially Dispensable for Its Activity

**FIGURE 3:**
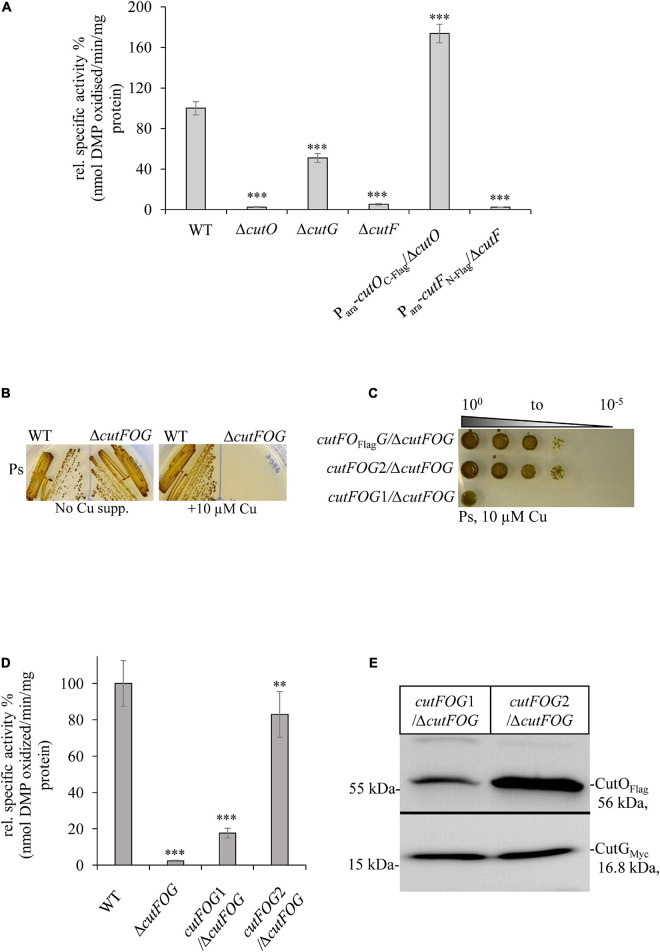
CutF but not CutG is essential for CutO activity. **(A)** CutO activity of WT and the Δ*cutO*, Δ*cutF*, and Δ*cutG* single mutant strains of *R. capsulatus*. The CutO activity was determined as described in the legend to Figure 1B. Epitope-tagged versions of *cutO* and *cutF* were expressed from an arabinose-inducible promoter from plasmid pRK415. The activity of WT was set to 100% and the relative activities of the indicated strains were calculated. Three independent experiments were performed with three technical replicates and the error bars reflect the standard deviation (*n* = 9). Statistical analyses were performed as described in Figure 1. (^∗^) refers to *p*- values ≤ 0.05; (^∗∗^) to *p*-values ≤ 0.01, and (^∗∗∗^) to *p*-values ≤ 0.001. **(B)** Cu sensitivity of the WT and the Δ*cutFOG* triple knock-out strain, grown under PS conditions and in the presence or absence of 10 μM CuSO_4_. **(C)** Spot assay for Cu sensitivity of the Δ*cutFOG* strain expressing differently tagged plasmid-borne copies of the *cutFOG-*operon. In *cutFO*_Flag_*G*, CutO was C-terminally Flag-tagged. In *cutFOG1* (*cutF_C__–_*_Strep_*O*_Flag_*G*_MycHis_) and *cutFOG2* (*cutF_*N*__–_*_strep_*O*_Flag_*G*_MycHis_), all three genes were tagged, but contained the Strep-tag either at the C-terminus (*cutFOG1*) or at the N-terminus (*cutFOG2*) of *cutF*. **(D)** CutO activities of the strains shown in panel **(C)**. Periplasmic fractions were isolated from the indicated strains, grown in MPYE medium supplemented with 10 μM CuSO_4_. 50 μg protein of the periplasmic fractions were used in the assay. Three independent experiments were performed with three technical repetitions and the error bars reflect the standard deviation (*n* = 9). The activity of WT was set to 100% and the relative activities of the other strains were calculated. Statistical analyses were as in Figure 1. (^∗^) refers to *p*-values ≤ 0.05; (^∗∗^) to *p*-values ≤ 0.01, and (^∗∗∗^) to *p*-values ≤ 0.001. **(E)** Immunoblot analyses of the Δ*cutFOG* strain harboring either the *cutFOG1* or *cutFOG2* constructs. 50 μg of soluble protein and α-Flag antibodies were used for the detection of CutO (upper panel) and 75 μg protein and α-Myc antibodies were used for detection of CutG (lower panel). Although Strep-tagged, CutF could not be detected in immunoblots in either construct, although the *cutFOG2* construct fully complemented the Δ*cutFOG* strain.

To correlate the observed Cu sensitivity phenotypes of the Δ*cutF*, Δ*cutO*, and Δ*cutG* mutants with their CutO activities, periplasmic fractions of the individual mutants were prepared and their CutO activities were measured spectrophotometrically using 2,6-DMP as a substrate (Figure 3A). In the absence of CutG, CutO activity was reduced to about 50% of the wild-type activity, whereas in the absence of CutF only very low CutO activity was observed (Figure 3A), demonstrating that while CutG is important, CutF is essential for CutO activity. The observed CutO activities in the Δ*cutF* and Δ*cutG* strains also correlated well with the observed Cu sensitivity phenotypes, suggesting that Cu resistance is directly linked to CutO activity.

Epitope tagged versions of *cutF*, *cutG* and *cutO* genes were cloned into an arabinose inducible plasmid and introduced into the respective mutants. A C-terminally Flag-tagged CutO variant fully rescued CutO activity in the Δ*cutO* strain, but an N-terminally Flag-tagged CutF variant did not rescue the Δ*cutF* mutant (Figure 3A). The Δ*cutF* strain was generated by an in-frame, markerless chromosomal deletion for avoiding any potential polar effect on *cutO* expression. Thus, the lack of complementation by an ectopic *cutF* copy, suggested that either the presence of the tag on CutF interfered with its activity, or that stable production of CutF required the integrity of the *cutFOG* operon.

This was analyzed by constructing a Δ*cutFOG* triple knock-out strain and testing its complementation by different *cutFOG*-encoding plasmids. As predicted from the phenotypes of the single mutants, the Δ*cutFOG* strain was highly sensitive to Cu (Figure 3B), but Cu sensitivity was rescued by the plasmid pRK-cutFO_Flag_G, which encoded the *cutFOG*-operon under control of its native promoter and a C-terminally Flag-tagged CutO variant (Figure 3C).

Subsequently, each gene within the operon was individually tagged to allow immunological identification of the corresponding gene products. For CutF, two variants were constructed, containing a Strep-tag either at the C-terminus (pRK-cutFOG1, *cutF_C__–_*_Strep_*O*_Flag_*G*_MycHis_) or at the N-terminus after the signal sequence cleavage site (pRK-cutFOG2 *cutF_*N*__–_*_Strep_*O*_Flag_*G*_MycHis_). Plasmid pRK-cutFOG2 fully rescued the Cu sensitive phenotype of the Δ*cutFOG* strain, while pRK-cutFOG1 did so only partially (Figure 3C). Analyses of the CutO activities of these strains showed that CutO activity was not detectable in the Δ*cutFOG* strain, but activity was fully restored by the presence of pRK-cutFOG2 (Figure 3D). In contrast, in the presence of pRK-cutFOG1 only about 15% of wild type activity was detected (Figure 3D).

Immune detection of the periplasmic fractions isolated from the Δ*cutFOG* strain carrying pRK-cutFOG1 or pRK-cutFOG2 revealed lower CutO levels in pRK-cutFOG1 containing cells, while the CutG levels were comparable (Figure 3E and Supplementary Figure 5). Thus, the addition of a Strep-tag to the conserved C-terminus of CutF apparently impaired its function, reducing the steady-state amounts of CutO and its activity. Despite the availability of N-terminally or C-terminally tagged variants, immune detection of CutF was not possible in strains expressing pRK-cutFOG1 or pRK-cutFOG2, in line with earlier unsuccessful attempts (Rademacher et al., 2012; Selamoglu et al., 2020).

**FIGURE 4:**
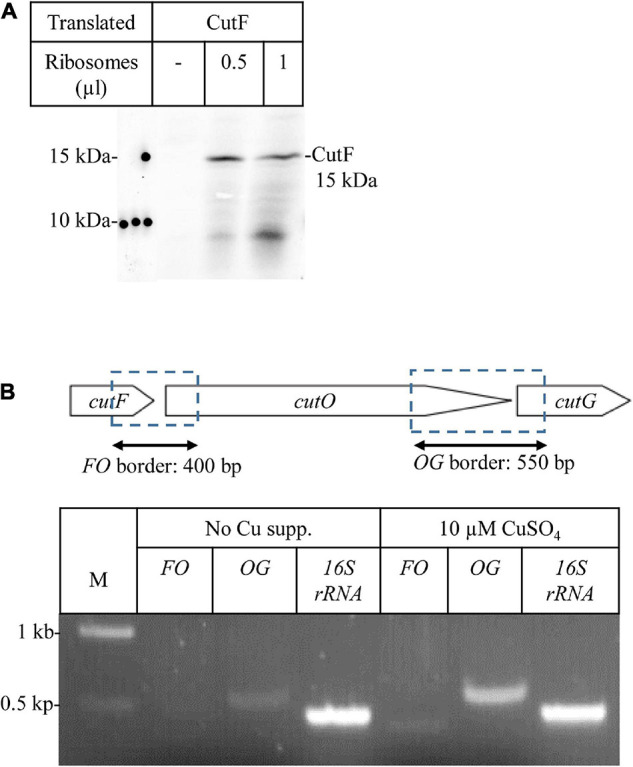
CutF is translated into protein **(A)** *In vitro* protein synthesis of CutF. After *in vitro* synthesis, samples were separated by SDS-PAGE, and the radioactively labeled bands were detected by phosphorimaging. Different volumes (μl) of purified *Escherichia coli* ribosomes were used for *in vitro* synthesis **(B)** Transcriptional analysis of the *cutFOG* operon. Physical map of the *cutFOG* operon (upper cartoon representation) indicating the DNA fragments emerging from RT-PCR (double arrow). RT-PCR analyses of *cutFOG* mRNA levels in *cutFOG2*/Δ*cutFOG* cells grown on MPYE without and with 10 μM Cu^2+^ supplementation. The 16S ribosomal RNA (450 bp) was used as an internal control. The intensities of the bands were determined by the *ImageJ* software. A negative control PCR omitting templates was performed in each case to check for DNA contamination (data not shown).

For exploring the molecular basis of this lack of CutF detection, an *E. coli in vitro* coupled transcription-translation system was used to confirm that CutF can be produced, as inferred by the genetic complementation assays using constructs maintaining the *cutFOG* operon integrity (Figure 3C). Indeed, *in vitro* translation confirmed the production of a protein of correct molecular mass corresponding to *cutF*_*N*__–Flag_ ORF (Figure 4A), further suggesting that the *cutF* product is either produced at very low levels or undergoes rapid degradation *in vivo*. A possible degradation of CutF would also explain the presence of the weaker approx. 10 kDa product that is detected after *in vitro* translation (Figure 4A). Next, appropriate RT-PCR experiments were performed to assess if the low level of CutF originates from its poor transcription, using total RNA extracted from pRK-cutFOG2/Δ*cutFOG* cells grown in the absence, or presence of 10 μM CuSO_4_ supplementation. Appropriate primer pairs (Supplementary Table 2) for the fragments overlapping the gene borders of either *cutFO* (400 bp) or *cutOG* (550 bp) were used for RT-PCR. Quantification of the amplified fragments by *ImageJ* revealed that the 3′ *cutOG* border region was amplified approx. six times more than the 5′ *cutFO* border region in the presence of Cu (Figure 4B). Internal controls using genomic DNA confirmed the specificity and efficiency of the selected primer pairs (Supplementary Figure 6). Assuming that *cutFOG* genes form a single transcription unit (Wiethaus et al., 2006), this result suggested that the stability of the 5′ end of the produced mRNA encompassing *cutF* is much lower than its 3′ end corresponding to *cutO* and *cutG*, in agreement with the low-level production of *cutF* translation product. It was previously shown that the *cutF–cutO* intergenic region contains a stem-loop structure that affects the stability of the *cutFOG* mRNA, playing a role in its post-transcriptional Cu response (Rademacher et al., 2012).

### The Conserved Putative Cu Binding Motif and the Proline Residues at the C-Terminus of CutF Are Essential for Its Function

**FIGURE 5:**
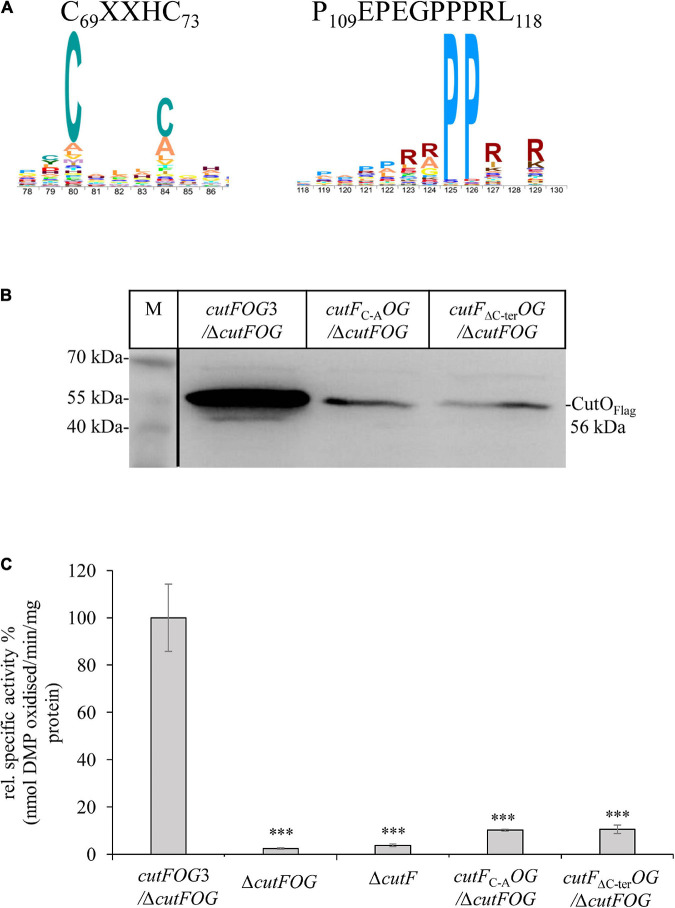
The conserved cysteine motif and the proline-rich C-terminus of CutF are essential for its function. **(A)** Sequence logo of the conserved CutF motifs. **(B)** Immunoblot analysis of the CutO proteins from the strains carrying the *cutF*_C–A_*OG* (Substitution of putative Cu binding C_69_XXXC_73_ motif by A_69_XXXA_73_) and *cutF*_Δ__C–ter_*OG* (truncation of C-terminal proline-rich region, P_109_EPEGPPPRL_118_) plasmids producing the CutF_C–A_ and CutF_Δ__C–ter_ mutant proteins, respectively. In both plasmids CutO was C-terminally Flag-tagged. 200 μg of soluble protein and α-Flag antibodies were used for the detection of CutO. 15% SDS-PAGE was used for separation. **(C)** CutO activities in the Δ*cutFOG* strain expressing either *cutFOG3* (*cutF_*N*__–_*_Flag_*O*_Flag_*G*_MycHis_) or the mutated *cutF*_C–A_*OG* or *cutF*_Δ__C–ter_*OG* variants. In all cases, CutO was C-terminally Flag-tagged. The Δ*cutFOG* and the single Δ*cutF* mutants served as an additional control. Strains were grown on MPYE medium in the presence of 10 μM CuSO_4_. 50 μg of the same soluble protein from the periplasmic fractions were used in the assay. Three independent experiments were performed with three technical repetitions and the error bars reflect the standard deviation (*n* = 9). Statistical analyses were performed as in Figure 1, using the *cutFOG3*/Δ*cutFOG* strain as reference. (^∗^) refers to *p*- values ≤ 0.05; (^∗∗^) to *p*-values ≤ 0.01, and (^∗∗∗^) to *p*-values ≤ 0.001.

The highly conserved putative Cu binding CXXXC motif in the central part of CutF, and the unusual proline-rich sequence surrounded with charged residues at its C-terminal end (Figure 5A), were probed by mutagenesis for their plausible functions using the pRK-cutFOG3 (*cutF*_*N*__–Flag_*O*_Flag_*G*_MycHis_) construct which maintains the *cutFOG* operon integrity. The C_69_XXH_72_C_73_ motif was changed to AXXHA, and the ten C-terminal amino acids (P_109_EPEGPPPRL_118_) of CutF were deleted, yielding the plasmids pRK-CutF_C–A_OG and pRK-CutF_Δ C–ter_OG, respectively. Expression of these CutF variants in the Δ*cutFOG* strain showed that both mutations significantly reduced the CutO levels (Figure 5B and Supplementary Figure 7), resulting in a ∼ 90% decrease of CutO activity, comparable to the CutO activity in the Δ*cutF* strain (Figure 5C). These data suggested that the conserved CXXXC motif, usually found in Cu chaperones, and the proline-rich sequence are essential for the function of CutF for CutO maturation and activity. Whether or not these CutO defects originate from a lack of proper Cu binding to CutF or a productive CutF-CutO interaction, remains to be further analyzed.

The availability of the mutant CutF variants allowed us to further assess the need for the structural integrity of the *cutFOG* operon by testing the CutO activity in the Δ*cutOG* strain carrying the pRK-cutF_C–A_OG plasmid. In this strain a functional *cutF* is expressed from the chromosome and functional *cutOG* are located on a plasmid. Similarly, the CutO activity of the Δ*cutFG* mutant carrying the pRK-cutFO_C__473__A_G plasmid with an inactive CutO copy (see below section “CutO Confers Cu Resistance by O2-Dependent and O2-Independent Mechanisms”) and intact copies of *cutFG* on the plasmid was analyzed. The latter strain contained an intact copy of *cutO* on the chromosome. The CutO activities in both strains were as low as in the Δ*cutO* or Δ*cutF* single mutants (Supplementary Figure 8), again supporting the need for the integrity of the *cutFOG* operon for CutO production, suggesting that transcription, translation or secretion of the *cutFOG* genes or their products are coupled.

### The Conserved Putative Cu Binding Motifs of CutG Are Not Required for CutO Activity

**FIGURE 6:**
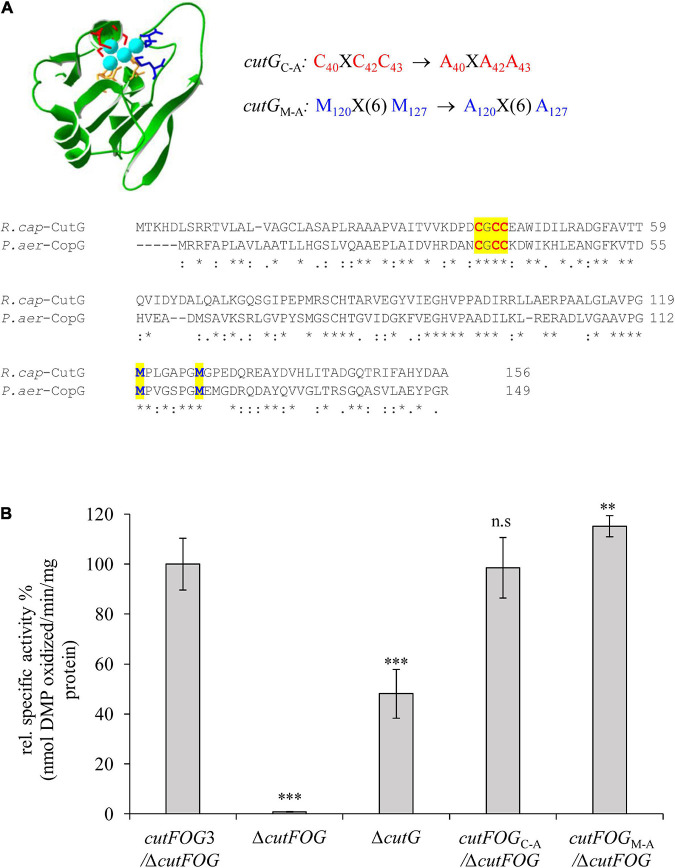
CutG is not essential for CutO activity **(A)** Model for the *R. capsulatus* CutG built on the X-ray structure of CopG from *Pseudomonas aeruginosa* (PDB ID: 6WIS) by the Swiss-Model software. The upper panel shows the conserved motifs and their substitutions. The lower panel shows the amino acid alignment between the *R. capsulatus* (*R. cap*) CutG and *P. aeruginosa* (*P. aer*) CopG. The conserved Cysteines and Methionines are highlighted in red and blue letters, respectively. **(B)** CutO activities of the indicated strains expressing *cutG*_C–A_ and *cutG*_M–A_. The periplasmic fractions were isolated from the strains grown on MPYE medium in the presence of 10 μM CuSO_4_. 50 μg total protein was used in the 2,6-DMP assay. The activity of the Δ*cutFOG* strain carrying the plasmid pRK-*cutFOG3* (*cutF_*N*__–_*_Flag_*O*_Flag_*G*_MycHis_) strain was set to 100% and the relative activities of the other strains were calculated. Three independent experiments were performed with three technical repetitions and the error bars reflect the standard deviation (*n* = 9). Statistical analyses were performed as in Figure 1, using the activity of the *cutFOG3*/Δ*cutFOG* strain as reference. (^∗^) refers to *p*- values ≤ 0.05; (^∗∗^) to *p*-values ≤ 0.01, and (^∗∗∗^) to *p*-values ≤ 0.001.

Mutagenesis was also performed on CutG by alanine replacements of the conserved Cys and Met residues of its C_40_XC_42_C_43_ and M_120_X(6)M_127_ motifs, respectively, using the pRK-cutFOG3 (*cutF*_*N–*__Flag_*O*_Flag_*G*_MycHis_) construct to yield the CutG_C–A_ and CutG_M–A_ variants (Figure 6A). Unlike the CutF mutations, neither of these CutG mutations significantly affected CutO activity (Figure 6B), in agreement with its dispensable role for CutO activity and maturation as well as for Cu tolerance, as already seen with a Δ*cutG* strain (Figure 2 and Supplementary Figures 2, 3). The higher CutO activities of the CutG mutants in comparison to the Δ*cutG* mutant suggests that either the putative Cu-binding motifs of CutG are dispensable for its function in CutO maturation or that possible maturation defects are compensated for by the increased production of the plasmid-encoded CutG variants.

### Bioinformatic Analysis of CutF-Like Proteins and Their Co-occurrence With Other Cu- Related Genes

Bioinformatic analyses were initiated to determine the distribution of CutF homologs in bacterial genomes. Likely due to the relatively small size of CutF (118 amino acids) and the low sequence conservation across homologs, searches for sequence-similarity using BLASTp against the UniProt database resulted in only a few significant hits. However, we observed that the proteobacterial *cutO* homologs are often located next to a gene encoding a small protein roughly the same length as CutF, containing a CXXXC motif, a putative signal peptide, and a C-terminal PP motif, similar to *R. capsulatus* CutF. Based on these features, we performed a rule-based approach to generate an initial set of CutF-like proteins encoded by genes neighboring copper oxidase genes. This initial set was then used to initiate a less-restrictive search for CutF-like proteins using iteratively built profile-HMMs. We reasoned that CutF-like proteins may also be in gene neighborhoods that lack MCO genes or may have some sequence divergence not represented by the initial set.

**FIGURE 7:**
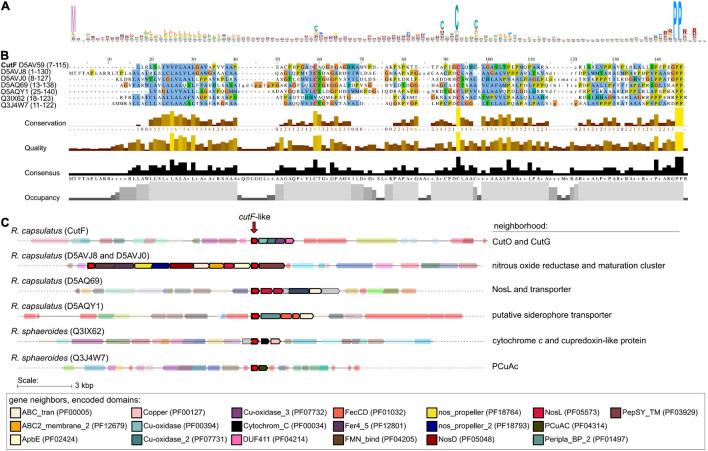
Identification of CutF-like proteins in publicly available sequenced genomes. **(A)** Sequence logo representing profile hidden Markov model (from the 10th iteration of jackhammer) used to identify CutF-like sequences. **(B)** Multiple sequence alignment as viewed with Jalview (Waterhouse et al., 2009) of identified CutF-like sequences from *R. capsulatus* and *Rhodobacter sphaeroides*. The color scheme used is that of clustalx, and the UniProt protein IDs are indicated as labels. The quantitative alignment annotations below the alignment displayed as histograms are automatically calculated by Jalview for each column. **(C)** Genomic context of identified CutF-like sequences from *R. capsulatus* and *R. sphaeroides*. “Neighborhood” refers to genes likely in an operon with the gene encoding the CutF-like protein. Genes are colored according to identified Pfam domains shown at the bottom of the figure.

**FIGURE 8:**
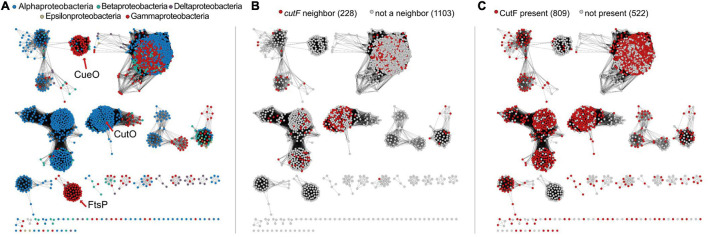
Sequence similarity networks of Cu-oxidase-like proteins. **(A)** network representing sequences containing Pfam domains PF00394, PF07731, and PF07732 from Uniprot-designated reference proteomes in the proteobacterial lineage. Nodes are colored according to the legend shown in each panel. The location of nodes representing CueO and FtsP from *E. coli* and CutO from *R. capsulatus* are indicated with red arrows. **(B)** Nodes are colored as to whether *cutF* is a neighbor of the Cu-oxidase-encoding gene. **(C)** Nodes are colored based on whether CutF is encoded in the genome or not. For panels **(B,C)**, numbers in parentheses on top of each panel represent the number of nodes in the network.

Using this approach, we identified nearly 3,000 CutF-like sequences in reference proteomes (The UniProt Consortium, 2019) from proteobacteria, including five CutF-like sequences of *R. capsulatus* and two of *Rhodobacter sphaeroides* species (Figure 7). As exemplified by these seven proteins, the iterative nature of our analysis captured proteins that do not have the CXXXC motif found in CutF, but do have a conserved cysteine-containing variation of this motif located in roughly the same region of the protein. The C-terminal PP motif is highly conserved among these proteins. The closest annotated HMM in the PFam database to the identified CutF-like proteins is DUF2946 (PF11162), which enclose small proteins with a C-terminal PP motif and a CXXC motif instead of the CXXXC motif in the central region. Intriguingly, DUF2946 was previously associated with Cu homeostasis through gene neighborhood analysis (Kenney and Rosenzweig, 2018). CutF-like proteins, as identified here in proteobacteria, are found in all classes of proteobacteria. There is an apparent enrichment for CutF-like proteins in alpha-proteobacteria (58%), but when taking into account the number of reference proteomes available for each class, there are roughly an equal amount of CutF-like proteins in alpha- and beta-proteobacteria, half as many in gamma-proteobacteria and a quarter as many in delta-proteobacteria (Figure 8).

Having identified a set of proteins with sequence characteristic analogous to CutF, we next asked to what extent are these proteins associated with homologs of CutO. To differentiate CutO proteins from other Cu oxidases, we built a sequence similarity network (Figure 8). We found that 40% of the genes corresponding to proteins from the CutO cluster are neighboring a gene encoding a CutF-like protein, and roughly 71% of proteins belonging to the CutO cluster are encoded by genomes that also encode a CutF-like protein. The high degree of genomic co-occurrence illustrated by these proteins, suggested that a functional link between CutO and CutF is not limited to *Rhodobacter*. In addition to *Rhodobacterales* (where there were 41 cases), gene proximity was also observed in *Rhizobiales* (31 cases) and *Rhodospirillales* (5 cases). Moreover, 55% of CutO-like proteins are encoded by genomes that contain genes for both the CutF and CutG proteins. In comparison, co-occurrence between CutF-like proteins and clusters containing CueO- (the *E. coli* multicopper oxidase) and FtsP- (cell division protein with a multicopper oxidase-like structure) like proteins is low, being only 8% and 2%, respectively (Figure 8 and Supplementary Figure 9). However, we did observe gene proximity with a CutF-like protein and Cu oxidase proteins from outside the CutO cluster in the alpha-proteobacterial lineages Caulobacterales (2), Parvularculales (1), and Sphingomonadales (43), the beta-proteobacterial lineages Burkholderiales (10), Neisseriales (1), and Nitrosomonadales (1), and the gamma-proteobacterial lienages Acidiferrobacterales (2), Alteromonadales (11), Cellvibrionales (9), Chromatiales (4), Enterobacterales (2), Oceanospirillales (11), Pseudomonadales (11), Thiotrichales (3), and Xanthomonadales (29).

Next, we focused our gene neighborhood analysis on the CutF-like proteins. Only 23% of CutF-like proteins are associated with Cu oxidase genes, suggesting that our analysis captured CutF-like proteins that could be involved in processes independent of a Cu oxidase. Based on the putative Cu-binding proteins identified by Andreini et al. (2008), 88% of the neighborhoods contained at least one gene encoding for a putative Cu-binding protein (Supplementary Table 3), such as nitrous oxide reductase, the Cu chaperones NosL, CopC, SenC, and PccA, as well as the Cu transporters CopB, CopD, and the Cus efflux system (Supplementary Table 3). These associations plus the presence of cysteine motifs suggest that CutF-like proteins are involved in Cu homeostasis, and that there are likely different subgroups interacting with different proteins.

### CutO Confers Cu Resistance by O_2_-Dependent and O_2_-Independent Mechanisms

**FIGURE 9:**
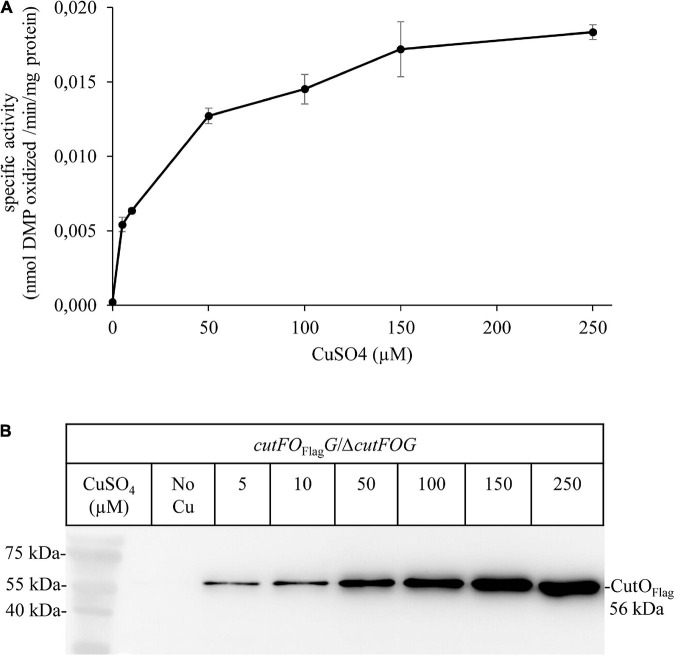
Activity and steady-state levels of CutO are Cu-dependent. **(A)** The activity of plasmid-encoded Flag-tagged CutO (*cutFO*_Flag_*G*) expressed in the Δ*cutFOG* strain, grown in MPYE medium supplemented with different CuSO_4_ concentrations. 50 μg protein of the periplasmic fractions were used for the assay. Shown are the mean values of three independent assays with three technical repetitions and the error bars reflect the standard deviation (*n* = 9). **(B)** For immunoblot analysis of the CutO_Flag_, approximately 40 μg of periplasmic proteins were separated on 15% SDS-PAGE and treated with anti-Flag antibodies.

An additional plasmid-borne CutFO_Flag_G variant was constructed in which the *cutFOG* expression is controlled by its native promoter to probe if the steady-state amounts and activities of CutO are influenced by the Cu concentration in the medium. This plasmid fully complemented the Δ*cutFOG* triple knock-out strain, and periplasmic extracts of these cells grown in the presence of different Cu concentrations were analyzed. The data showed a Cu-concentration dependent increase of the CutO activity (Figure 9A) that coincided with increased CutO protein levels (Figure 9B and Supplementary Figure 10A). This is in line with the Cu-induced increase of the CutO levels observed by cuproproteome analyses of *R. capsulatus* (Selamoglu et al., 2020) and with studies using in-frame *cutO*-lacZ fusions (Rademacher et al., 2012).

**FIGURE 10:**
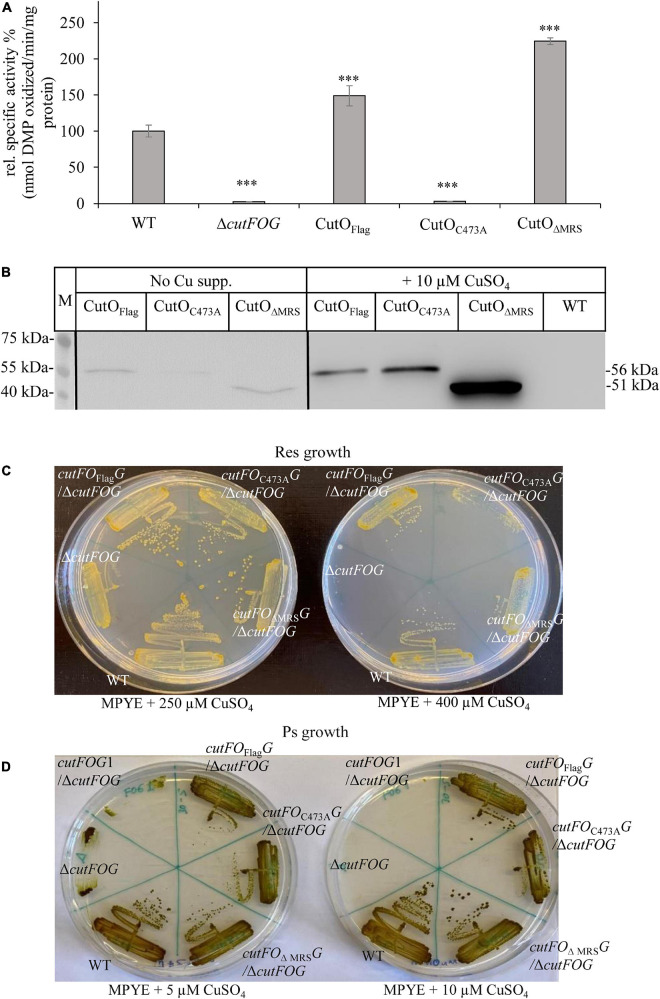
Activity, steady-state levels, and Cu sensitivity of different CutO mutants. **(A)** CutO activity of the CutO mutant variants CutO_C__473__A,_ carrying a mutation in the T1Cu binding motif, and CutO_Δ__MRS_, in which the methionine-rich segment (MRS) was deleted, was determined by using 50 μg protein of the periplasmic fraction isolated from strains grown on MPYE medium in the presence of 10 μM CuSO_4_. Three independent experiments were performed with three technical repetitions and the error bars reflect the standard deviation (*n* = 9). The activity of the WT strain was set to 100% and the relative activities of the other strains were calculated. Statistical analyses were performed as in Figure 1, using the activity of the WT strain as reference. (^∗^) refers to *p*- values ≤ 0.05; (^∗∗^) to *p*-values ≤ 0.01, and (^∗∗∗^) to *p*-values ≤ 0.001. **(B)** Immunoblot analysis of Flag-tagged wild type CutO and its mutant variants grown in the presence and absence of CuSO_4_ supplement. After isolation of the periplasmic fraction, 50 μg protein were separated on 12% SDS PAGE and treated with anti-Flag antibodies. **(C)** Respiratory (Res) and **(D)** photosynthetic (PS) growth of *R. capsulatus* strains carrying a plasmid born copy of CutO_Flag_ or its mutant variants (CutO_C__473__A_ and CutO_Δ__MRS_) on MPYE medium supplemented with different amounts of CuSO_4_. The *cutFOG*1/Δ*cutFOG* strain carrying the *cutF_C__–_*_Strep_*O*_Flag_*G*_MycHis_ construct was used as an additional control for Ps growth.

Multicopper oxidases like CutO use O_2_ as an electron acceptor and thus are catalytically active only in the presence of O_2_ (Solomon et al., 2008). Therefore, it was surprising that the Δ*cutO* strain was highly Cu sensitive even under anaerobic conditions (Figure 2). This suggested that CutO might confer Cu resistance not just by the O_2_-dependent oxidation of Cu^1+^ to the less toxic Cu^2+^, but also by another O_2_-independent mechanism. Two CutO variants, CutO_C__473__A_ and CutO_Δ__MRS_, produced from the plasmid pRK-cutFO_Flag_G were constructed to further analyze this finding. In the CutO_C__473__A_ variant, the Cys473 ligand of the T1 Cu center was replaced by alanine, which is expected to inactivate CutO. In the CutO_Δ__MRS_ variant, the MRS, which accommodates the Cu ions Cu5, Cu6, and Cu7 in the *E. coli* homolog CueO was deleted, which is expected to reduce the ability of CutO to bind Cu (Kataoka et al., 2007; Singh et al., 2011). Both plasmids were individually transferred into the Δ*cutFOG* strain, and cells were tested for CutO activity, CutO levels, and Cu sensitivity (Figure 10).

The CutO_C__473__A_ mutant had no detectable CutO activity just like the Δ*cutFOG* strain, but in the CutO_Δ__MRS_ mutant, the CutO activity was approx. 2-fold higher than wild-type activity (Figure 10A). This is likely due to the increased accumulation of this CutO variant as indicated by immuno-detection using anti-Flag antibodies (Figure 10B and Supplementary Figure 10B). In addition, the absence of the MRS might favor access of bulky organic substrates to the catalytic site of CutO. This was shown for *E. coli* CueO, where the deletion of the MRS led to a 10% reduction of the cuprous oxidase activity, while it increased the oxidation of alternative substrates, like phenolic components such as 2,2’-Azino-bis (3-ethylbenzothiazoline-6-sulfonic acid) or 2,6-DMP (Kataoka et al., 2007). The MRS was therefore proposed to provide a specificity filter to ensure preferentially Cu^1+^ oxidation (Roberts et al., 2003). When testing the Cu sensitivity profile on enriched medium supplemented with 250 μM CuSO_4_ under similar Res growth conditions, both mutant variants showed Cu resistance levels comparable to those conferred by the plasmid-borne wild-type CutO (Figure 10C), while the Δ*cutFOG* strain failed to form single colonies. When the Cu concentration was increased to 400 μM, the Δ*cutFOG* strain was completely unable to grow, while cells expressing either the CutO_C__473__A_ variant or the CutO_Δ__MRS_ variant still grew, although growth was reduced in comparison to wild-type CutO (Figure 10C). Moreover, when the same experiment was repeated under anaerobic-PS conditions, the Δ*cutFOG* strain was significantly growth-impaired already at 5 μM Cu and did not grow at 10 μM Cu. In contrast, both of the two CutO variants grew almost like the MT1131 wild-type strain with native CutO, or a Δ*cutFOG* strain complemented with endogenous CutFOG levels (Figure 10D). The observation that the CutO_C__473__A_ variant, which has no O_2_-dependent Cu oxidase activity, also confers Cu resistance further indicated that CutO-mediated Cu resistance also involves an O_2_-independent step. The O_2_-dependent and O_2_-independent Cu resistance mechanisms of CutO seem to be set apart in the catalytically inactive CutO_C__473__A_ mutant variant, as this mutant still provides Cu tolerance even in the absence of O_2_. Hence, a dual function of CutO in Cu resistance emerges: (1) O_2_-dependent enzymatic reduction of Cu^1+^ to the less toxic Cu^2+^, and (2) protein-mediated sequestration of Cu, reducing the total Cu concentration. The latter possibility is supported by the presence of additional Cu sites in the MRS of *E. coli* CueO (Singh et al., 2011) and by the observation that neither the inactivation of the T1 site nor the deletion of the MRS abolishes Cu resistance (Figure 10D).

## Discussion

**FIGURE 11:**
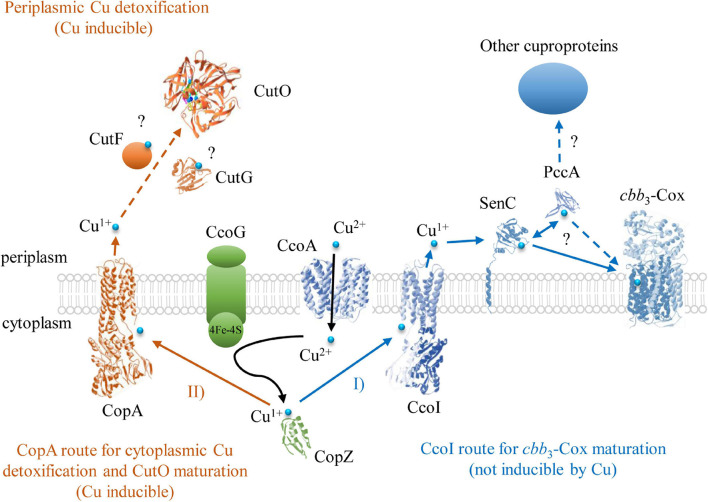
The Cu detoxification and cuproenzyme biogenesis pathways for *cbb*_3_-Cox and CutO in *R. capsulatus*. Two Cu export routes to the periplasm have been identified so far in *R. capsulatus*. One of them is through the P_1__B_-type ATPase CcoI that exports Cu^1+^ to the periplasm for *cbb*_3_-Cox assembly. In the periplasm, Cu is bound to SenC (or alternatively to PccA) and subsequently inserted into the catalytic subunit CcoN of *cbb*_3_-Cox. The other route is via the P_1__B_-type ATPase CopA, which has a lower affinity/higher turnover rate for Cu, and exports excess cytosolic Cu^1+^ to the periplasm for CutO maturation and subsequent oxidation to Cu^+2^. In addition to the CopZ-CopA Cu delivery pathway for CutO maturation, alternative Cu supply pathways for CutO likely exist in *R. capsulatus*, but have not been characterized yet. Structures were retrieved from the protein database with the following IDs: 3RFU (CopA and CcoI), 6WIS (CopG for CutG), 3OD3 (CueO for CutO), 1K0V (CopZ), 3WDO (YajR for CcoA), 4WBR (ScoI/SenC), 2K70(PCuAC/PccA), 5DJQ (*cbb*_3_-Cox), and depicted using Swiss-PdbViewer.

In Gram-negative bacteria, P_1__B_-type ATPases CopA and the CopZ-like chaperones have been identified as major determinants for Cu detoxification of the cytoplasm (Gonzalez-Guerrero and Arguello, 2008; Argüello et al., 2013; Utz et al., 2019). The multicopper oxidase CueO (CutO) provides a similar function for the periplasm by oxidizing Cu^1+^ to the less toxic Cu^2+^ and likely by sequestering excess Cu via the MRS (Petersen and Moller, 2000; Grass and Rensing, 2001; Fernandes et al., 2007; Singh et al., 2011; Novoa-Aponte et al., 2019). In many, but not all bacteria, the expression of genes determining cytoplasmic and periplasmic Cu tolerance, like *copA*, *copZ*, and *cueO*, are coordinated by the transcriptional regulator CueR (Outten et al., 2000; Stoyanov et al., 2001; Peuser et al., 2011). In the current study, we investigated the maturation of the cuproenzyme CutO with respect to its assembly and the mechanism by which it provides Cu resistance. The better-known multi-step machinery for Cu insertion into *cbb*_3_-Cox directed us to inquire if any known component(s) of this machinery is also used for the assembly of CutO. The results indicated that CutO activity is not affected significantly by the lack of the known *cbb*_3_-Cox assembly factors CcoA, SenC, and PccA. The latter components are critical under low Cu availability whereas Cu detoxification, including CutO induction, requires higher Cu concentration, in line with these findings. Among the remaining components, a small but reproducible decrease (∼25% of wild-type activity) was seen in the Δ*ccoG* strain. Mutant strains lacking the Cu^2+^ reductase CcoG are defective in *cbb*_3_-Cox assembly and also exhibit Cu sensitivity (Marckmann et al., 2019), possibly due to the decreased CutO activity in a Δ*ccoG* strain as seen here. Under aerobic conditions, Cu is present primarily as Cu^2+^, while most Cu chaperones and P_1__B_-type ATPases involved in exporting Cu to the periplasm only bind Cu^+^. Hence, the elimination of CcoG might result in reduced cytoplasmic Cu^1+^ quota, leading to less Cu^1+^ loaded CopZ and subsequently to reduced Cu^1+^ supply to both the CopA and CcoI P_1__B_-type ATPases (Utz et al., 2019; Figure 11). The Cu^2+^-reducing activity of CcoG and the Cu-transfer activity of CopZ are the only so far identified nexuses between Cu resistance and *cbb*_3_-Cox assembly in *R. capsulatus*.

The deletion of *copZ* and *copA* decreased the periplasmic CutO activity significantly (65 and 60% of wild-type, respectively), indicating that the CopZ-CopA dependent cytoplasmic detoxification system provides at least a portion of Cu^1+^ required for CutO maturation. However, the residual CutO activity in the absence of CopZ or CopA also indicates that alternate pathway(s) for supplying Cu to CutO exists in *R. capsulatus*, which need to be further explored. In Gram-negative bacteria, outer membrane proteins, like OmpC or ComC, contribute to Cu tolerance by so far unknown mechanisms (Kershaw et al., 2005; Mermod et al., 2012; Giachino and Waldron, 2020) and it is possible that Cu for CutO maturation in *R. capsulatus* can also be provided by Cu uptake from the environment. In any event, partial dependency of CutO activity on the cytoplasmic CopZ-CopA Cu detoxification pathway implies that compartmental Cu detoxification processes are coordinated to ensure efficient Cu homeostasis (Outten et al., 2001; Peuser et al., 2011; Rademacher et al., 2012; Pezza et al., 2016; Figure 11). Intriguingly, although the effects of CopA and CopZ on CutO activity were similar, their effects on Cu tolerance (inhibitory concentration ∼20 μM for Δ*copA* and ∼200 μM CuSO_4_ for Δ*copZ*) were about tenfold different (Utz et al., 2019). This suggests that in this species an alternative CopZ-independent pathway is able to convey Cu to CopA, which delivers Cu to the periplasm for CutO maturation. In contrast to *P. aeruginosa*, which contains two CopZ paralogs (CopZ1 and CopZ2) (Novoa-Aponte et al., 2019), a second *copZ* is not detectable in the *R. capsulatus* genome and the CopZ-independent Cu delivery pathway to CopA remains to be characterized.

Under both aerobic and anaerobic Ps conditions, the catalytically inactive CutO_C__473__A_ variant still provided Cu resistance, although to a lesser extent than the native CutO. This is intriguing because the strictly conserved T1 Cu site is absent in this mutant due to the mutation of the C-terminal HCH motif (Roberts et al., 2002; Augustine et al., 2010). In addition, under anaerobic conditions, CutO is assumed to be inactive due to the absence of O_2_ as an electron acceptor. How CutO supports Cu resistance in the absence of the T1 Cu and O_2_ is not obvious. Considering that additional Cu binding sites have been identified in the MRS region of MCOs (Fernandes et al., 2007; Singh et al., 2011), a likely possibility is that CutO contributes to Cu resistance not only by Cu(I) oxidation but also by sequestering Cu via its secondary Cu binding sites, including MRS. This is also supported by the observation that deleting the MRS of CutO attenuated Cu resistance as compared to wild type or the CutO_C__473__A_ variant of CutO. The high cellular content of the inactive Cut_C__473__A_ variant also indicates that the T1Cu, although essential for activity, is not required for CutO stability.

The precursor of CutO has, like its *E. coli* homolog CueO, a Tat signal peptide, which is often found in proteins that are transported across the membrane in a folded and inactive state (Kudva et al., 2013). In the case of *E. coli* CueO, it has been shown that a signal peptide-deficient CueO variant produced in the cytoplasm contained no bound Cu (even under Cu supplemented conditions) and that externally added Cu can be readily inserted *in vitro* to yield an active protein (Stolle et al., 2016). These findings together with the observed dependence of CutO maturation on the CopZ-CopA Cu export pathway and the co-localization of CutO with the two putative periplasmic Cu binding proteins CutF and CutG within the *cutFOG* operon suggest that maturation of active CutO likely occurs in the periplasm.

The experimental results of *cutFOG* knockout strains and the mutant variants of CutF and CutG proteins showed that CutF, but not CutG, is essential for CutO assembly. Indeed, mutating the conserved Cys residues of CutG, which ligate the two Cu ions, or mutating the two Met residues, which constitute the ligands for another Cu atom (Hausrath et al., 2020), showed that this protein is dispensable for CutO maturation. This could be due to the presence of a functionally redundant protein in the *R. capsulatus* periplasm. Alternatively, it has been suggested that *P. aeruginosa* CopG, which is homologous to CutG, might act as a Cu oxidoreductase that balances the Cu^1+^/Cu^2+^ equilibrium, and thereby contributes to Cu tolerance (Hausrath et al., 2020). Thus, CutG might be primarily required as a back-up Cu defense system.

In general, due to the toxicity of Cu, cells use chaperones with very high Cu affinity to prevent the availability of free Cu in cell compartments (Banci et al., 2010; Novoa-Aponte et al., 2019, 2020), and to convey Cu to its destination using specific protein-protein interactions (Banci et al., 2009). Biogenesis of membrane-integral and periplasmic cuproenzymes involves a large variety of specialized periplasmic Cu chaperones, in comparison to the limited variety of cytoplasmic chaperones. Clearly, a need for direct and specific protein-protein interactions emerges between the Cu donor chaperones and Cu accepting cuproproteins (e.g., SenC and PccA for *cbb*_3_-Cox, NosL for NosZ, and CueP for superoxide dismutase), which drive the biogenesis of a multitude of extracytoplasmic cuproproteins (Andrei et al., 2020). Although our data clearly demonstrate the essential role of CutF in CutO maturation, its exact function is currently unknown. It could act as a Cu chaperone that takes part in the Cu^1+^ transfer from CopA to CutO, in line with its essential CXXXC motif that could ligate Cu^1+^ with a trigonal planar geometry, similar to the Cu binding motif of SenC (Figure 11). A periplasmic function of CutF is also supported by its predicted cleavable signal sequence, which suggests a post-translational translocation into the periplasm. In *E. coli*, the periplasmic chaperone CusF accepts Cu^1+^ directly from the Cu-bound form of CopA by specific interaction upon ATP hydrolysis (Padilla-Benavides et al., 2014). A decrease in Cu^1+^ transfer efficiency was observed upon mutating the extracellular loops of CopA, or the electropositive surface of CusF (Padilla-Benavides et al., 2014). Similarly, CutF with its Cu chaperone-like Cu-binding motif and its genome-wide co-localization with other genes encoding cuproproteins as indicated by bioinformatic analyses may function as a periplasmic Cu^1+^ chaperone for CutO maturation. At the C-terminus, CutF contains a conserved proline-rich region. These regions preferentially adopt a type II helical conformation that facilitates protein-protein interactions, as shown e.g., for TonB or OmpA (Williamson, 1994). In CutF, this region may provide a structural domain for interactions with CutO and/or CopA.

Remarkably, bioinformatic analyses documented a more general role for CutF and CutF-like proteins as important members of the Cu homeostasis networks and cuproprotein biogenesis. CutF-like proteins seem widespread in proteobacteria and are more often found in alpha-proteobacterial species. On average, two CutF-like genes were found per genome, and typically in distinct gene neighborhoods, suggesting that these paralogs may have evolved to function in different Cu-trafficking processes. The corresponding genes are often found in gene neighborhoods with Cu chaperones, such as NosL, CopC, CusF, SenC, and PccA, suggesting that in those organisms, CutF-like proteins are involved in Cu homeostasis.

However, different to other periplasmic Cu chaperones, which are easily detectable and present in μM concentrations in *Rhodobacter* cells (Trasnea et al., 2018), CutF appears to be of very low abundance and is basically undetectable in *Rhodobacter* (Rademacher et al., 2012; Selamoglu et al., 2020). Still, the extremely low amount of CutF is both necessary and sufficient for full CutO activity, provided that *cutF* is located in *cis*, next to *cutO* conserving the structural integrity of *cutFOG* operon. Examination of *cutF* expression using a coupled *in vitro* transcription-translation system showed that its ORF can indeed be translated if transcribed, and related RT-PCR results indicated that its low cellular level may stem from its mRNA stability. Although *cutFOG* comprises a single transcription unit (Wiethaus et al., 2006), the 5′ end of this mRNA corresponding to *cutF* seems less abundant than the remainder covering *cutO* and *cutG*, in agreement with the higher steady-state levels of CutO and CutG in cells. The 40 bp long *cutF–cutO* intergenic region contains a stem-loop structure that possibly affects the stability of this mRNA. Mutagenesis of the *cutF–cutO* intergenic region indicated that it encodes for a Cu-responsive mRNA element that is essential for the Cu-dependent expression of *cutO* and *cutG* (Rademacher et al., 2012). Future studies addressing the exact function of CutF would require detecting CutF and monitoring its cellular localization.

## Data Availability Statement

The original contributions presented in the study are included in the article/Supplementary Material, further inquiries can be directed to the corresponding author/s.

## Author Contributions

YÖ, CB-H, ND, AA, JR, FD, and H-GK contributed to the design of the study, the acquisition, analysis, and interpretations of the data. All authors contributed to writing the article and approved the submitted version.

## Conflict of Interest

The authors declare that the research was conducted in the absence of any commercial or financial relationships that could be construed as a potential conflict of interest.

## Publisher’s Note

All claims expressed in this article are solely those of the authors and do not necessarily represent those of their affiliated organizations, or those of the publisher, the editors and the reviewers. Any product that may be evaluated in this article, or claim that may be made by its manufacturer, is not guaranteed or endorsed by the publisher.

## Footnotes

1
http://efi.igb.illinois.edu/efi-est/
