# Supplementary material for: Maturation of Rhodobacter capsulatus Multicopper Oxidase CutO Depends on the CopA Copper Efflux Pathway and Requires the cutF Product
Source: Front Microbiol. 2021 Sep 8;12:720644. doi: 10.3389/fmicb.2021.720644 (PMC8456105; doi:10.3389/fmicb.2021.720644)
Supplement: Supplementary file 2 [file Data_Sheet_1.pdf]

Table S1. Strains and plasmids used in this work.

**Strain or plasmid**	**Description**	**Phenotype**	**Reference**
Strains<br><br>***E. coli***			
HB101	F^- Δ(*gpt-proA*)62 *leuB6 supE44 ara*-14 *galK2 lacY*1<br>Δ(*mcrC-mrr*) *rpsL20* (Str^R) *xyl-5 mtl-1recA*13	Str^r	(Sambrook and Russell, 2001)
S17-1	*recA pro hsdR, RP4(Tc::Mu, Km::Tn7)*		(Simon et al., 1983)
DH5-α	*supE44 ΔlacU169* ***(****Φ80lacZΔM15****)*** *hsdR17 recA1 endA1 gyrA96 thi-A1 thi-1 relA1*		(Taylor et al., 1993)
R. capsulatus			
[a]MT1131	*crtD121*	wild type (Rif^r)	(Zannoni et al., 1980)
Y262	GTA overproducer		(Yen et al., 1979)
CW2	Δ(*ccoI::Spe*)	Spe^r	(Koch et al., 2000)
CW5	Δ(*ccoG::Spe*)	Spe^r	(Koch et al., 2000)
SE15	Δ(*cutO::Kan*)	Kan^r	(Ekici et al., 2012)
SE24	Δ(*copA::Kan*)	Kan^r	(Ekici et al., 2014)
Δ*copZ*	Δ(*copZ::Gen*)		(Utz et al., 2019)

LS01	Δ(*senC*), seamless in frame deletion		(Swem et al., 2005)
IT1	Δ(*pccA::Kan*)	Kan^r	(Trasnea et al., 2016)
SE8	Δ(*ccoA::Spe*)	Spe^r	(Ekici et al., 2012)
YO15	Δ(*ccoI::Spe*), Δ(*copA::Kan*)	Kan^r, Spe^r	This work
YO-ΔcutF	Δ(*cutF*(*rcc02111*)) seamless in frame deletion	In-frame, markerless	(Selamoglu et al., 2020)
YO-ΔcutG	Δ(*cutG*(*rcc02109::Gm*))	Gm^r	(Selamoglu et al., 2020)
YO-ΔcutFO	Δ (*cutF*(*rcc02111*)), Δ(*cutO::Kan*)	Gm^r	This work
YO-ΔcutOG	Δ(*cutO::Kan*), Δ(*cutG*(*rcc02109::Gm*)),	Gm^r	This work
YO-ΔcutFG	Δ (*rcc02111*), Δ(*cutG*(*rcc02109::Gm*))	In-frame, markerless	This work
YO-ΔcutFOG	Δ (*rcc02111*), Δ(*rcc03065-rcc03067::Gm*), Δ(*cutO::Kan*)	Gm^r	This work
Plasmids			
pRK2013	Conjugation helper	Kan^r	(Ditta et al., 1985)
pRK415	Broad host-range vector	Tet^r	(Ditta et al., 1985)
pZDJ	Puc promoter replaced with *tetA* promoter on the suicide plasmid pZJD29a	*sacB*, Gm^R	(Brimacombe et al., 2013)
pRS1	pPET19 based containing pBR322 ori, Rop, T7RNAP under EM7 promoter, LacI	Amp^r	(Jauss et al., 2019)

pYO-Δ02109	*Δ(rcc02109::Gm)* on pRK415	Tet^r, Gm^r	(Selamoglu et al., 2020)
pYO-Δ02111Su	*Δ(rcc02111)* on pZDJ; in-frame deletion of *rcc02111* without its first 4 and last 4 codons.	Gm^r	(Selamoglu et al., 2020)
pRK415-copZ gentamicin	*Δ(copZ::Gen)* on pRK415	Tet^r, Gm^r	(Utz et al., 2019)
pRK-CopA2::Kan	*Δ(copA2::Kan)*	Kan^r	(Ekici et al., 2014)
pYO-cutOFlag	only *cutO*$_{Flag}$ under P_{BAD} in pRK415	Tet^r	This work
pRKara-cutF$_{N\text{-}Flag}$	only *cutF*$_{N\text{-}Flag}$ under P_{BAD} in pRK415	Tet^r	This work
pRK-cutFOG1	*cutF*$_{C\text{-ter Strep}}$, *cutO*$_{Flag}$ and *cutG*$_{MycHis}$ with promoter and terminator regions of *cutFOG* operon on pRK415	Tet^r	This work
pRK-cutFOG2	*cutF*$_{N\text{-ter Strep}}$, *cutO*$_{Flag}$ and *cutG*$_{MycHis}$ with promoter and terminator regions of *cutFOG* operon on pRK415	Tet^r	This work
pRK-cutFO$_{Flag}$G	*cutF*, *cutO*$_{Flag}$ and *cutG* with promoter and terminator regions of *cutFOG* operon cloned on pRK415	Tet^r	This work
pRK-cutFO$_{C473A}$G	C473A substitution in *cutO*$_{Flag}$ with promoter and terminator regions of *cutFOG* operon cloned on pRK415	Tet^r	This work
pRK-cutFO$_{\Delta MRS}$G	Δ 41 aa Methionine Rich Segment (MRS) of *cutO*$_{Flag}$ with promoter and terminator regions of *cutFOG* operon cloned on pRK415	Tet^r	This work
pRK-cutFOG3	*cutF*$_{N\text{-ter Flag}}$, *cutO*$_{Flag}$ and *cutG*$_{MycHis}$ with promoter and terminator regions of *cutFOG* operon on pRK415	Tet^r	This work
pRK-cutF$_{C\text{-}A}$	substitution of conserved Cys to Ala of CutF ($C_{69}LQHC_{73}$ to $A_{69}LQHA_{73}$) on pRK-FOG3	Tet^r	This work

pRK-cutF$_{\Delta C\text{-ter}}$	truncation of c-terminus Pro rich region of CutF (ΔP_{109}EPEGPPPRL_{118}: 10 aa) on pRK-FOG3	Tet[r]	This work
pRK-cutG$_{C\text{-}A}$	substitution of conserved Cys to Ala of CutG ($C_{40}XC_{42}C_{43}$ to $A_{40}XA_{42}A_{43}$) on pRK-FOG3	Tet[r]	This work
pRK-cutG$_{M\text{-}A}$	substitution of conserved Met to Ala of CutG ($M_{120}X_{(6)}M_{127}$ to $A_{120}X_{(6)}A_{127}$) on pRK-FOG3	Tet[r]	This work
pRS-cutF	*cutF*$_{N\text{-}Flag}$ ORF cloned to pRS1 for in vivo and in vitro expression	Amp[r]	This work

[a] *R.capsulatus* strain MT1131 is derived from SB1003 in multiple steps, as described in Zannoni *et al*. (1980): first a Ps-deficient mutant (TL1) was obtained using tetracycline suicide, then its *crtD* derivative was constructed by GTA cross to yield MT113, and then its Ps-proficient derivative was obtained via a second GTA cross.

REFERENCES

Brimacombe, C.A., Stevens, A., Jun, D., Mercer, R., Lang, A.S., and Beatty, J.T. (2013). Quorum-sensing regulation of a capsular polysaccharide receptor for the *Rhodobacter capsulatus* gene transfer agent (RcGTA). *Mol Microbiol* 87**,** 802-817.

Ditta, G., Schmidhauser, T., Yakobson, E., Lu, P., Liang, X.W., Finlay, D.R., Guiney, D., and Helinski, D.R. (1985). Plasmids related to the broad host range vector, pRK290, useful for gene cloning and for monitoring gene expression. *Plasmid* 13**,** 149-153.

Ekici, S., Turkarslan, S., Pawlik, G., Dancis, A., Baliga, N.S., Koch, H.-G., and Daldal, F. (2014). Intracytoplasmic copper homeostasis controls cytochrome c oxidase production. *mBio* 5**,** e01055-01013.

Ekici, S., Yang, H.H., Koch, H.G., and Daldal, F. (2012). Novel Transporter Required for Biogenesis of cbb(3)-Type Cytochrome c Oxidase in Rhodobacter capsulatus. *Mbio* 3**,** 1-11.

Jauss, B., Petriman, N.A., Drepper, F., Franz, L., Sachelaru, I., Welte, T., Steinberg, R., Warscheid, B., and Koch, H.G. (2019). Noncompetitive binding of PpiD and YidC to the SecYEG translocon expands the global view on the SecYEG interactome in Escherichia coli. *Journal of Biological Chemistry* 294**,** 19167-19183.

Koch, H.G., Winterstein, C., Saribas, A.S., Alben, J.O., and Daldal, F. (2000). Roles of the ccoGHIS gene products in the biogenesis of the cbb(3)-type cytochrome c oxidase. *Journal of Molecular Biology* 297**,** 49-65.

Sambrook, J., and Russell, D.W. (2001). *Molecular Cloning: a laboratory manual.* Cold Spring harbor: Cold Spring harbor Laboratory Press.

Selamoglu, N., Önder, Ö., Öztürk, Y., Khalfaoui-Hassani, B., Blaby-Haas, C.E., Garcia, B.A., Koch, H.-G., and Daldal, F. (2020). Comparative differential cuproproteomes of Rhodobacter capsulatus reveal novel copper homeostasis related proteins. *Metallomics* 12**,** 572-591.

Simon, R., Priefer, U., and Puhler, A. (1983). A Broad Host Range Mobilization System for Invivo Genetic-Engineering - Transposon Mutagenesis in Gram-Negative Bacteria. *Bio-Technology* 1**,** 784-791.

Swem, D.L., Swem, L.R., Setterdahl, A., and Bauer, C.E. (2005). Involvement of SenC in assembly of cytochrome c oxidase in Rhodobacter capsulatus. *J Bacteriol* 187**,** 8081-8087.

Taylor, R.G., Walker, D.C., and Mcinnes, R.R. (1993). Escherichia-coli host strains significantly affect the quality of small-scale plasmid DNA preparations used for sequencing. *Nucleic Acids Research* 21**,** 1677-1678.

Trasnea, P.-I., Utz, M., Khalfaoui-Hassani, B., Lagies, S., Daldal, F., and Koch, H.-G. (2016). Cooperation between two periplasmic copper chaperones is required for full activity of the cbb3-type cytochrome c oxidase and copper homeostasis in Rhodobacter capsulatus. *Molecular microbiology* 100**,** 345-361.

Utz, M., Andrei, A., Milanov, M., Trasnea, P.I., Marckmann, D., Daldal, F., and Koch, H.G. (2019). The Cu chaperone CopZ is required for Cu homeostasis in Rhodobacter capsulatus and influences cytochrome cbb3 oxidase assembly. *Mol Microbiol* 111**,** 764-783.

Yen, H.C., Hu, N.T., and Marrs, B.L. (1979). Characterization of the gene transfer agent made by an overproducer mutant of *Rhodopseudomonas capsulata*. *J Mol Biol* 131**,** 157-168.

Zannoni, D., Prince, R.C., Dutton, P.L., and Marrs, B.L. (1980). Isolation and Characterization of a Cytochrome c_2-Deficient Mutant of *Rhodopseudomonas-capsulata*. *Febs Letters* 113**,** 289-293.

Table S2. Primers used in this work (5' to 3' direction).

Gm-F	5-AAGCTTGTCGACCCGATCTGAGC-3
Gm-R	5-TCTAGAACTAGTGGATCCCCCG-3
02111Su-F1	5-TACGCCAAGCTTGCATGCCTGCAGGATCGAGCTGTGGCAGGTGATGCGC-3
02111Su-R1	5-CAGAATCCGCATGGGAGGAGCATGGC-3
02111Su-F2	5-CCATGCTCCTCCCATGCGGATTCTGCCGCCCCGCCTCTGATCTTCGCC-3
02111Su-R2	5-AAACGACGGCCAGTGAATTCGAGCTGGCAGCGGCACCACCGGGTTGCC-3
02109-R1	5-AAAAATGGCTCAGATCGGGTCGACAAGCTTAAGGCACCCGGCGACAAGGGCC-3
02109-F2	5-TTCCCGGCCGGGGGATCCACTAGTTCTAGACTGATCACGGCGGACGGGCAAACC-3
02109-R2	5-AAGCTTGCATGCCTGCAGGTCGACTGTTCTAACCTTAGGCCGCCAGAC-3
1F-92NOQ	5-AGTGAATTCGAGCTCGGTACATGCATAATGTGCCTGTCAAATGG-3
cutO-F	5-GGGCTAACAGGAGGAATTAACCATG ACTCAGCTTTCCCGCCGCGGC-3
cutO-R	5-AAGCTTGCATGCCTGCAGGTCGACTTCACTTGTCATCGTCGTCCTTGTAGTCGGCGCTGACGACGAATTCGGTC-3
2111cl-F	5-GGGCTAACAGGAGGAATTAACCATGCGGATTCTGTGCGCCCTGC-3
2111cl-R	5-AAGCTTGCATGCCTGCAGGTCGACtTCAATGATGATGATGATGATGGTCGACGGCGCTATTCAGATCCTCTTCTGAGATG AGTTTTTGTTCGAGGCGGGGCGGCGGCCCCTCCG-3
cutTer-R	5-TGCATGCCTGCAGGTCGACtAATAGTCTCTTTGGCCTGCTGCCG-3
cutFopr-cterR	5-TCACTTTTCGAACTGCGGATGGCTCCACGCCGAGCCGCCGAGGCGGGGCGGCGGCCCCTC-3
cutOopr-F	5-CATCCGCAGTTCGAAAAGTGATCTTCGCCACGTTCCCATTCTTG-3
cutOopr-R	5-TCACTTGTCATCGTCGTCCTTGTAGTCGGCGCTGACGACGAATTCGGTC-3
cutGopr-F	5-AAGGACGACGATGACAAGTGAGGGGGAAACGAATATGACCAAGC-3
cutGopr-R	5-GCAGGGCGCCGCAGTCACGGTTCAATGATGATGATGATGATGGTCGACGGCGCTATTCAGATCCTCTTCCGAGATGAGC TTCTGTTCGGCCGCGTCGTAGTGGGCAAAG-3
cutFopr-NterR	5-CGCCGAGCCGCCCTTTTCGAACTGCGGATGGCTCCACGCGGCGACCACGGGCGCAACC-3
cutF+Oopr-F	5-TCGAAAAGGGCGGCTCG GCGCCGGAGGCCTGTCCGCATCCC -3
cutO(c473a)-F	5-GCGCACCACATGGGCCATCTTGCGACC-3
cutO(c473a)-R	5-TCACTTGTCATCGTCGTCCTTGTAGTCGGCGCTGACGACGAATTCGGTCATCATGCCGGTCGCAAGATGGCCCATGTGG TGCGCATGCAGCATCCAGGGC-3
cutOΔMRS-R	5-CGCCGTGCCGCCACTGACCGTCTC-3
cutOΔMRS-F	5-CGGTCAGTGGCGGCACGGCGGGCGGCGATCTGAACGATTACGAATTCGAC
CutF(Flag)-F	5-GACTACAAGGACGACGATGACAAGGGCGGCTCGGCGCCGGAGG-3
cutF(Flag)-R	5-TCATCGTCGTCCTTGTAGTCCGCGGCGACCACGGGCGCAACC-3

cutF(C-A)-F	5-GCGCTGCAGCATGCGCTGGGCGCCTCGCTGACGCCCG-3
cutF(C-A)-R	5-CCCAGCGCATGCTGCAGCGCGCCTTCCGGCGCGAAGGTGGTGG-3
cutF(Δ-Cter)-F	5-CTGCTTTCCTGATCTTCGCCACGTTCCCATTCTTGAACCG-3
cutF(Δ-Cter)-R	5-GCGAAGATCAGGAAAGCAGCGCCAGCCGCAACGGAACC-3
cutG(C-A)-F	5-GCGGGCGCGGCGGAGGCCTGGATCGATATTCTGCGC-3
cutG(C-A)-R	5-CAGGCCTCCGCCGCGCCCGCATCGGGGTCCTTGACGACAGTGATC-3
cutG(M-A)-F	5-GCGCCGCTTGGCGCCCCCGGCGCGGGCCCCGAGGATCAACGCGAGGC-3
cutG(M-A)-R	5-CGCGCCGGGGGCGCCAAGCGGCGCGCCCGGCACCGCCAGCCCCAG-3
pRS1-F	5-GCGGCCGCGAGCTCGTCGACCTCGAGTAGC-3
pRS1-R	5-CATGGGGTATATCTCCTTCTTAAAGTTAAACAAAATTATTTC-3
pRS1cutF-F	5-TTAAGAAGGAGATATACCCCATGCGGATTCTGTGCGCCCTGCTTCG-3
pRS1cutF-R	5-GTCGACGAGCTCGCGGCCGCTCAGAGGCGGGGCGGCGGCCCCTCC-3
cutFO(rtPCR)-F	5-ACCGTCGCCCAAGACCACCACC-3
cutFO(rtPCR)-R	5-ACCGGCCCTGCGCATCCAAAAGG-3
cutOG(rtPCR)-F	5-CGACGGGCGCAGCTGGGATAACC-3
cutOG(rtPCR)-R	5-GGATGCCGCTTTGCCCCTTGAGC-3
16SrRNA-F	5-ATATTCGGAGGAACACCAGTGGC-3
16SrRNA-R	5-CAGAGTGCCCAACTGAATGATGG-3

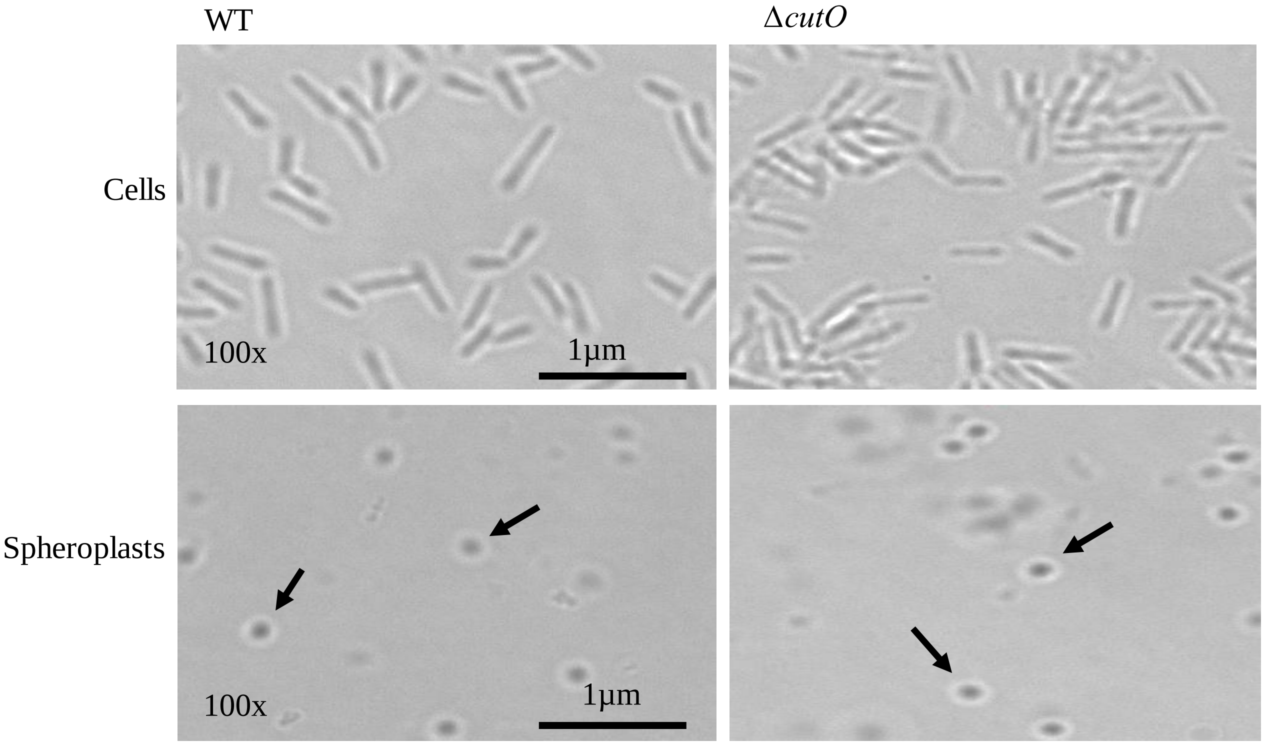

Figure S1. Monitoring spheroplast formation by microscopy. *R. capsulatus* WT and Δ*cutO* cells (upper part) and spheroplast formation of them (lower part) were observed at 100 x magnification with numerical aperture 1.4 (OLYMPUS, model: BX51). Spheroplasts are indicated by arrows.

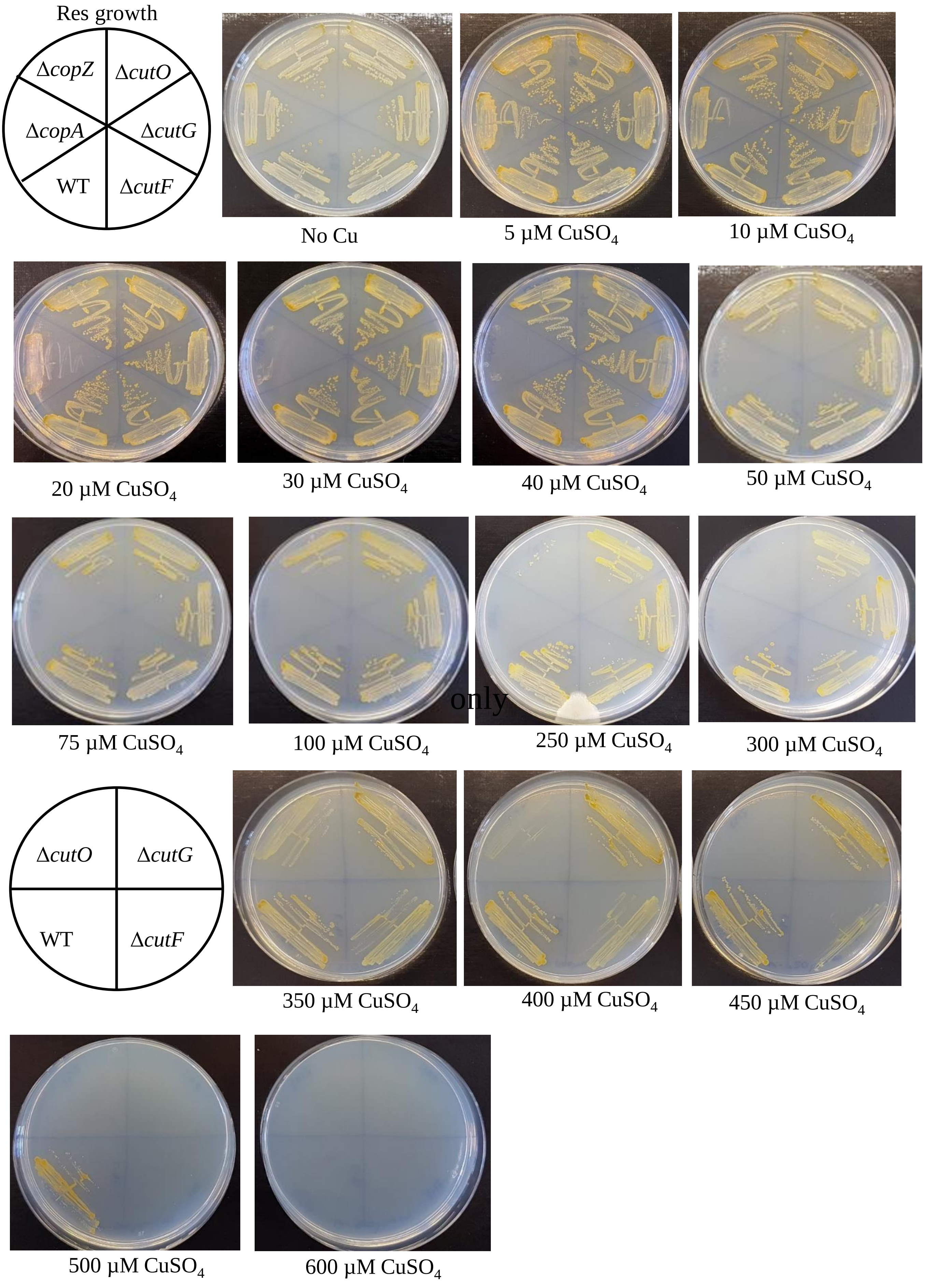

Figure S2. Respiratory (Res) growth of WT and *cutFOG* knockout strains (Δ*cutF*, Δ*cutO*, and Δ*cutG*) on MPYE medium supplemented with 5 µM to 600 µM $CuSO_4$ at 35°C for 3-4 days. Δ*copA* and Δ*copZ* mutants were used as a control.

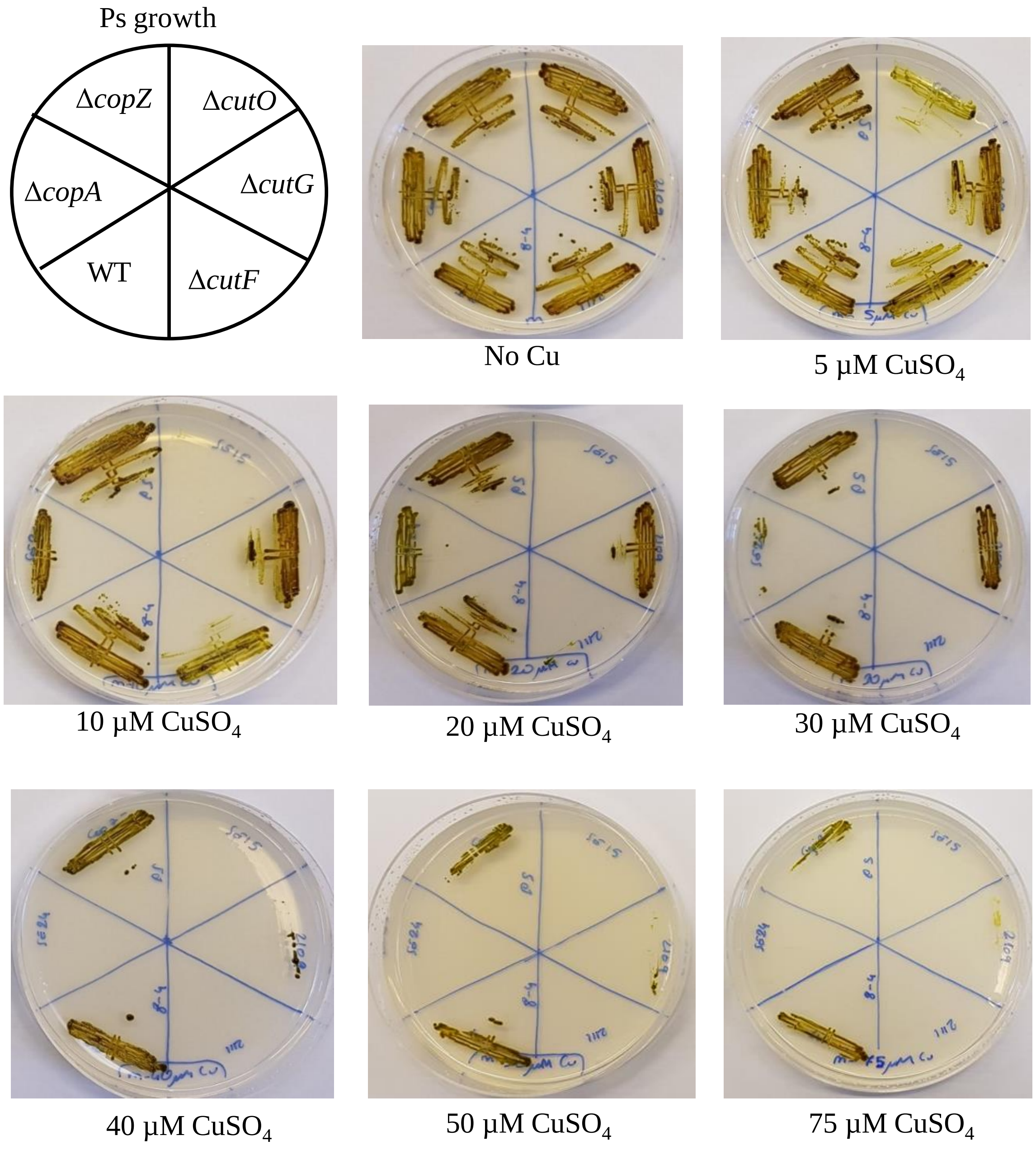

Figure S3. Photosynthetic (PS) growth of WT and *cutFOG* knockout strains (Δ*cutF*, Δ*cutO*, and Δ*cutG*) on MPYE medium supplemented with 5 µM to 75 µM $CuSO_4$ under saturating light intensity in anaerobic jars for 3-4 days. Δ*copA* and Δ*copZ* mutants were used as a control.

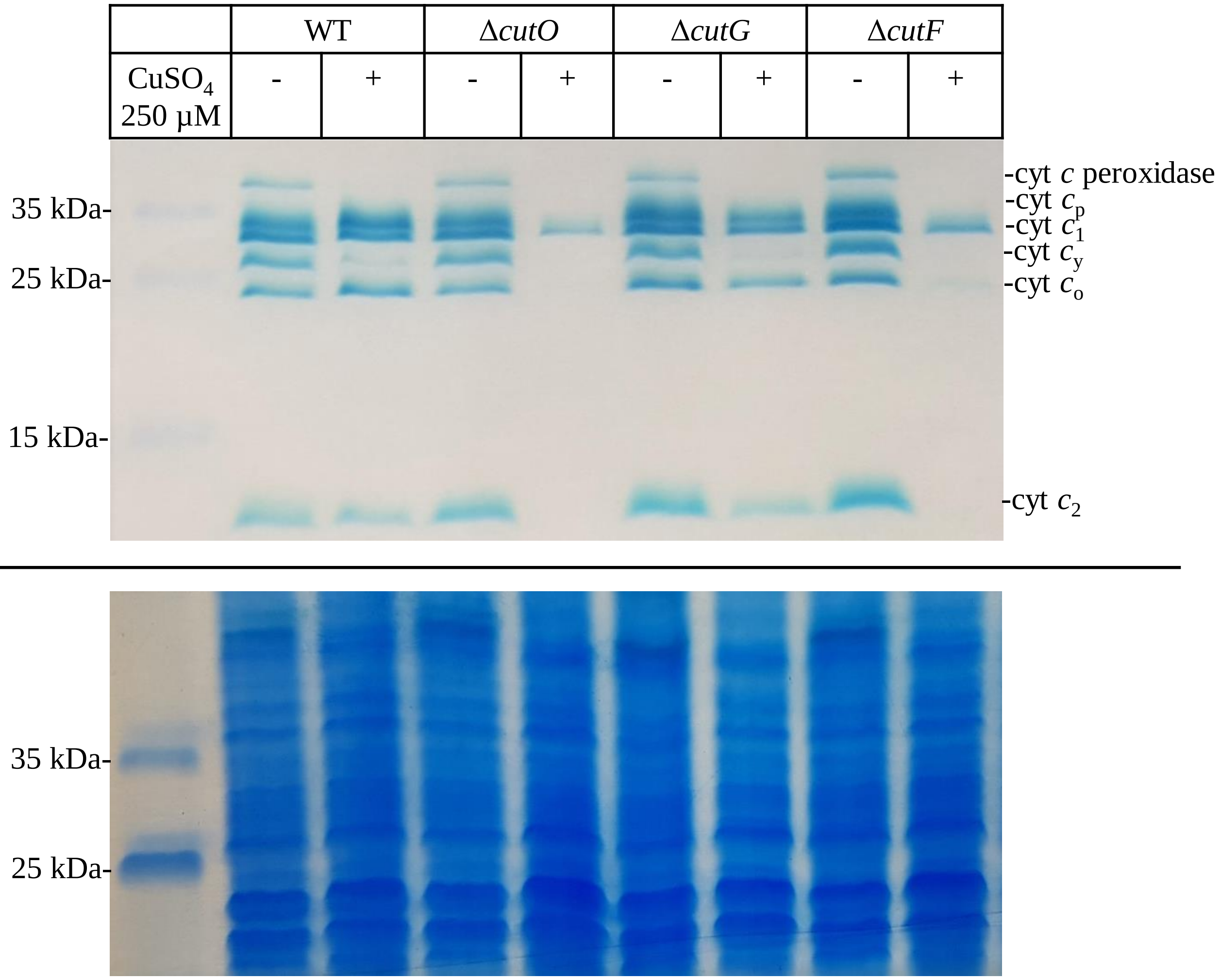

Figure S4. Loading control of Fig. 2C (upper panel). The duplicate of the same gel was stained with Coomassie brilliant blue (lower panel).

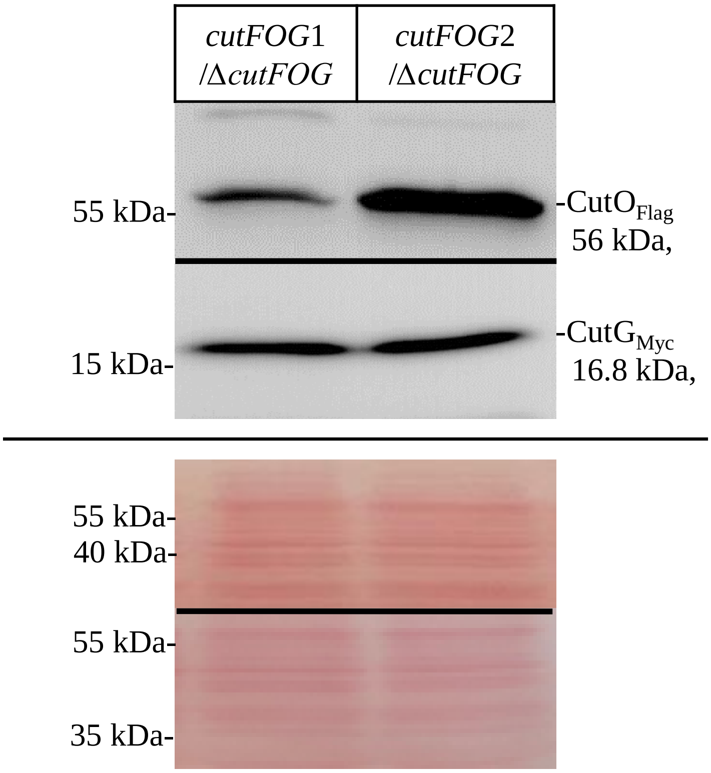

Figure S5. Loading control of Fig. 3E (upper panel). The same membranes were stained with Ponceau solution (lower panel).

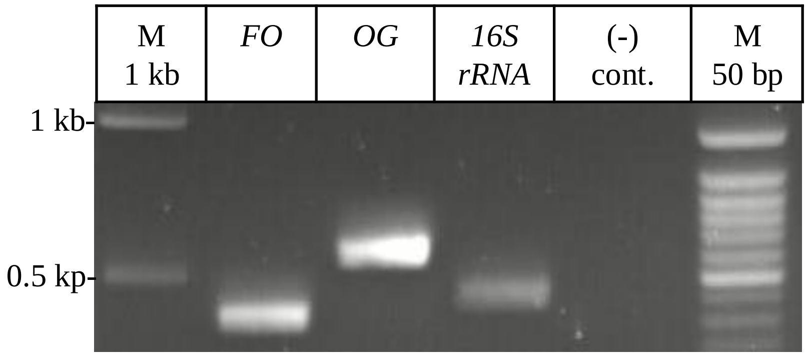

Figure S6. The efficiency of primers used for RT-PCR (Fig. 4B) were controlled by using the 60 ng chromosomal DNA from the WT MT1131 strain. PCR was performed by using the Q5® High-Fidelity DNA Polymerase following the manufacturer protocol. In negative control, the primers were not included in reaction mixture. The fragments *FO* and *OG* were efficiently produced at the expected sizes.

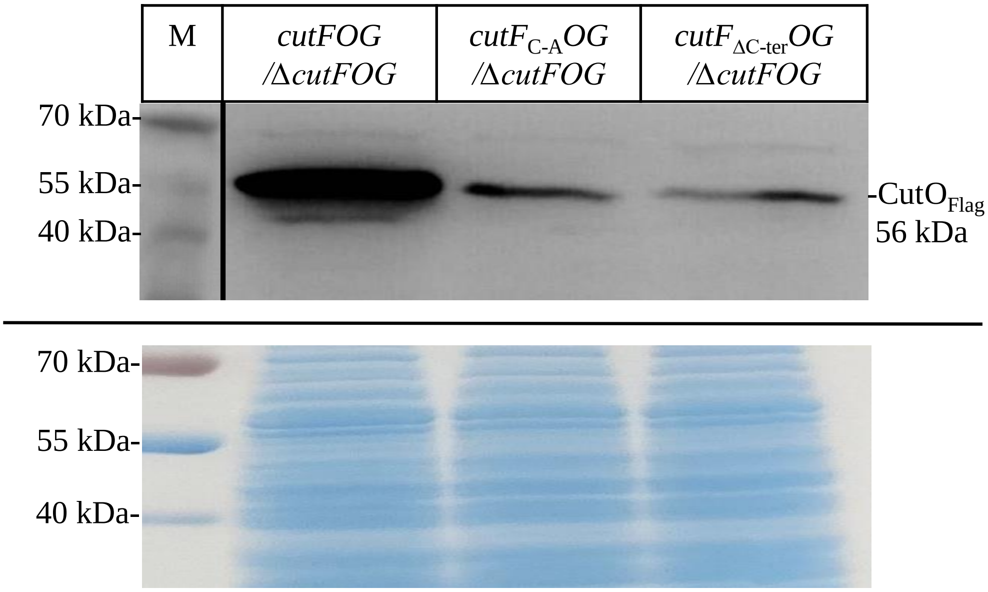

Figure S7. Loading control of Fig. 5B (upper panel). The duplicate of the gel was stained with Coomassie brilliant blue (lower panel).

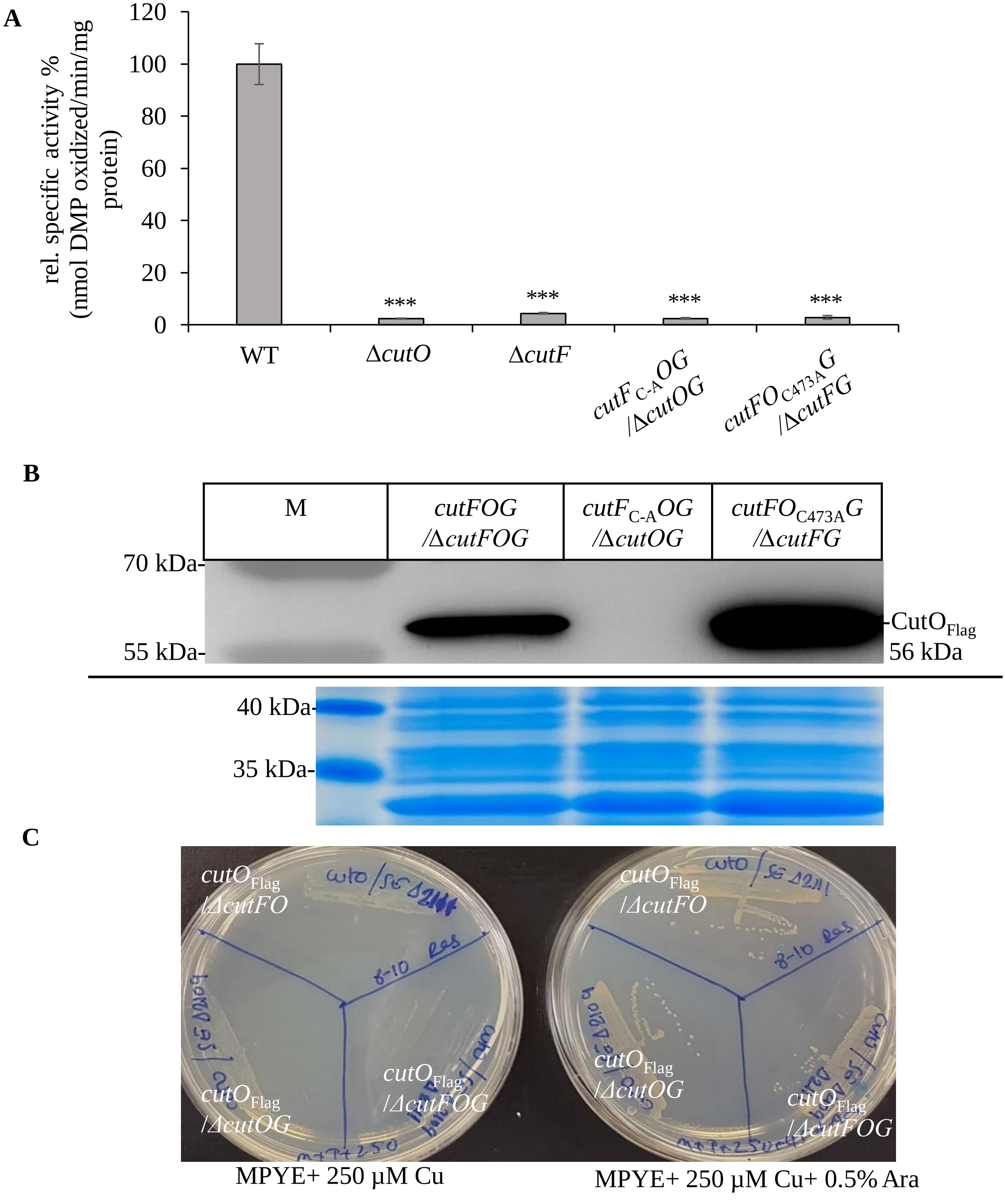

Figure S8. (A) CutO activity of *cutF*$_{\text{C-A}}$*OG/ΔcutOG* and *cutFO*$_{\text{C473A}}$*G/ΔcutFG* strains grown on MPYE supplement with 10 µM $CuSO_4$. WT and Δ*cutF* were used as an control. The CutO activity was determined as described in the legend to Figure 1B. Two independent experiments were performed with three technical repeats and the activity of wild-type was set to 100%. The error bars reflect the standard deviation (n=6). Statistical analyses were performed with the Satterthwaite corrected unpaired two-sided Student t-test, using the activity of the wild type. (*) refers to p- values ≤0.05; (**) to p-values ≤0.01, and (***) to p-values ≤0.001. (B) Immunoblot analysis of *cutFOG/ΔcutFOG, cutF*$_{\text{C-A}}$*OG/ΔcutOG* and *cutFO*$_{\text{C473A}}$*G/ΔcutFG* strains. After isolation of the periplasmic fraction, 50 µg protein were separated on 12-15% gradient SDS PAGE and treated with anti-Flag antibodies as described in Materials and Methods. The duplicate of the gel was stained with Coomassie brilliant blue for loading control (lower panel. (C) Cu sensitivity assay of $\text{CutO}_{\text{Flag}}$ under the arabinose inducible promoter in the Δ*cutOG,* Δ*cutFO* and Δ*cutFOG* background. In the presence of arabinose $\text{CutO}_{\text{Flag}}$ complements the strains transferred.

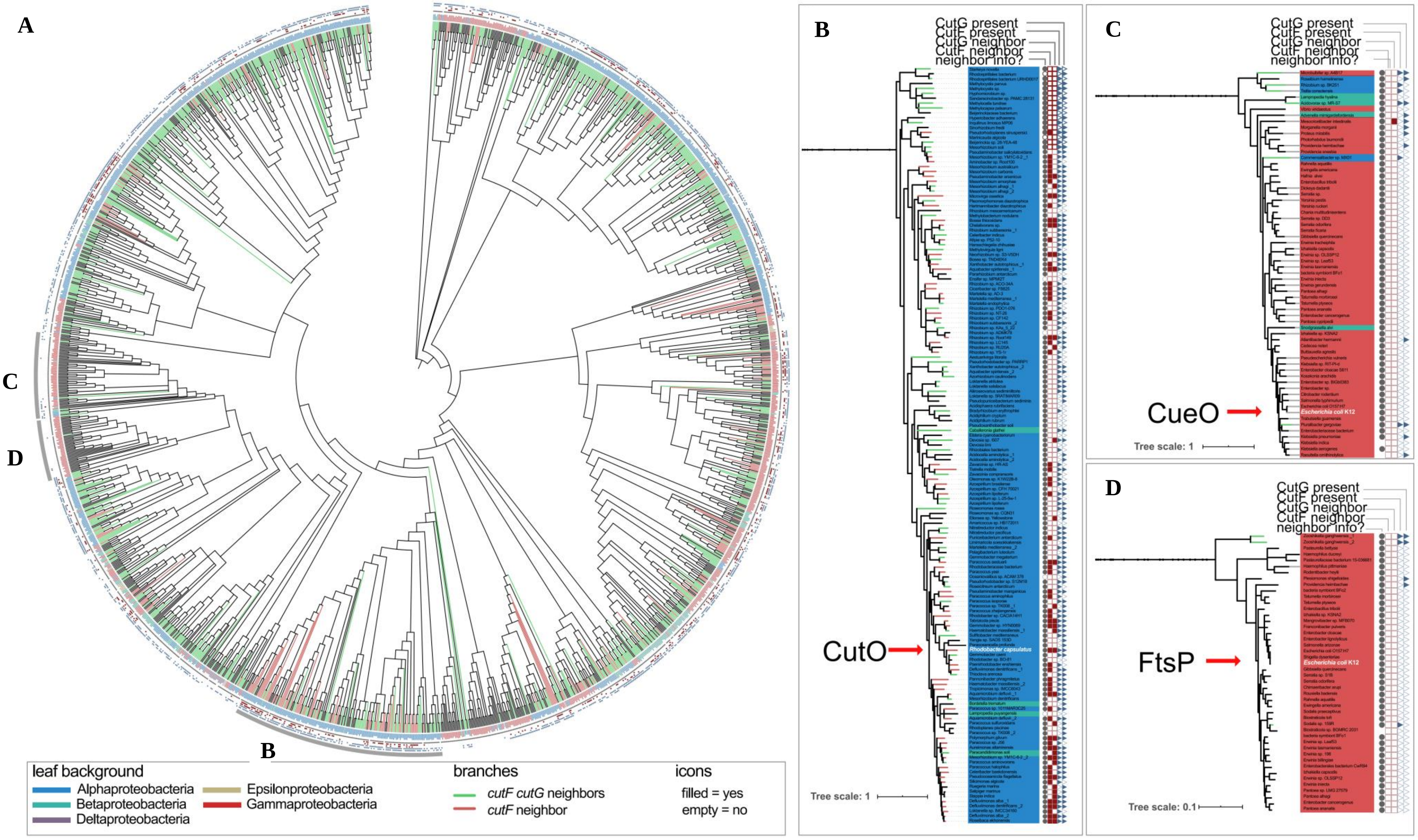

Figure S9. Phylogenetic reconstruction of Cu-oxidase-like proteins from Proteobacteria. (A) approximate maximum likelihood tree. Branch length is ignored. Grey bars correspond to sub-trees in panels B-D. (B) the clade containing CutO from *R. capsulatus*. (C) the clade containing CueO from *E. coli*. (D) the clade containing FtsP from *E. coli*. For panels B-D, the presence of CutF and/or CutG encoded in each genome is given to the right. The phylogenetic tree can be viewed at [https://itol.embl.de/tree/2418444149452551603891702.](https://itol.embl.de/tree/2418444149452551603891702)

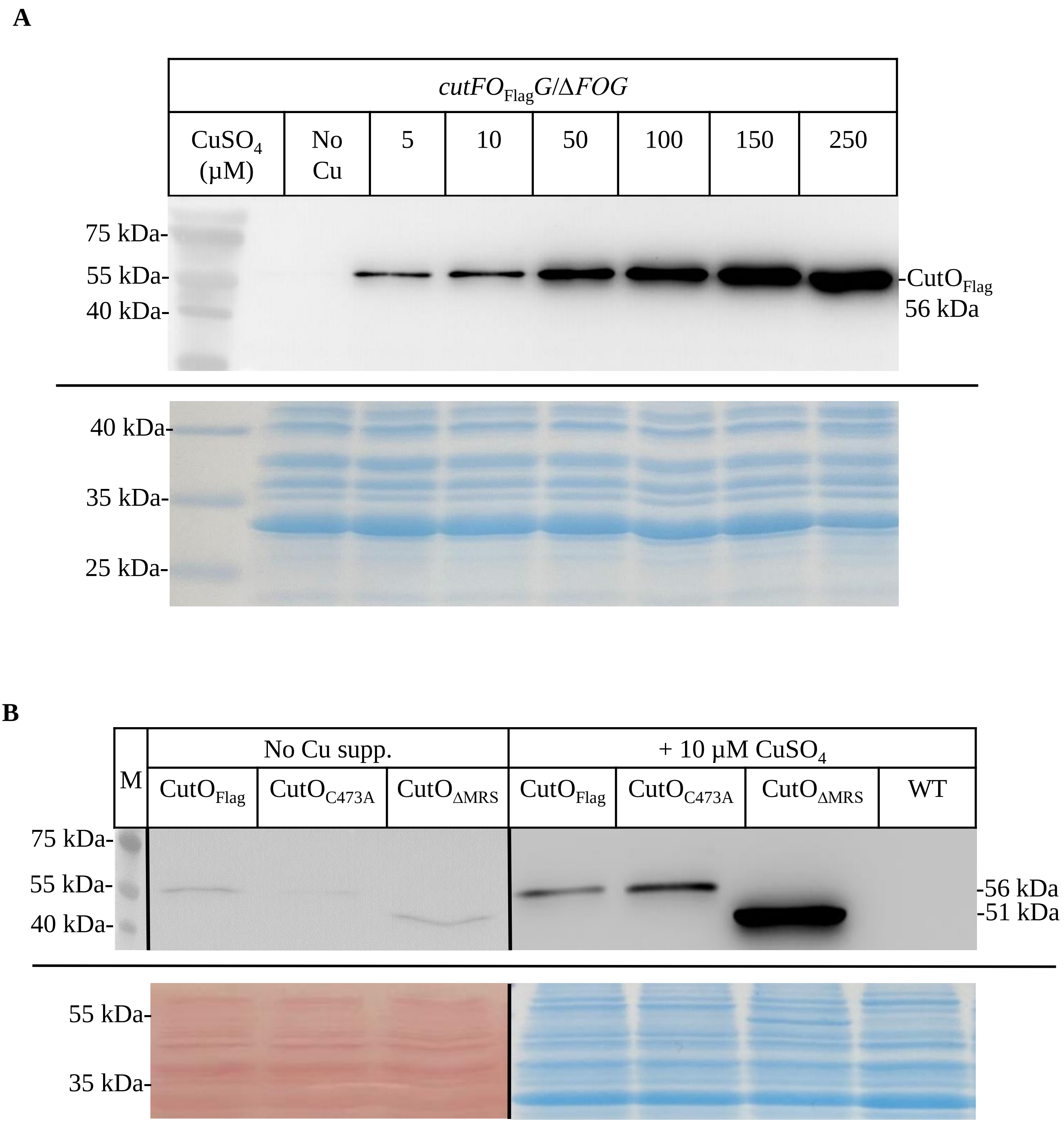

Figure S10. (A) Loading control of Fig. 9B (upper panel). The duplicate of the same gel was stained with Coomassie brilliant blue (lower panel). (B) Loading control of Fig. 10B (upper panel). The same membrane was stained with Ponceau solution for the No Cu supp. Samples (lower panel, left), and the duplicate of the same gel was stained with Coomassie brilliant blue for the samples grown in the presence of 10 µM supp. Cu (lower panel right).
